# Effective dose to adult patients from 338 radiopharmaceuticals estimated using ICRP biokinetic data, ICRP/ICRU computational reference phantoms and ICRP 2007 tissue weighting factors

**DOI:** 10.1186/2197-7364-1-9

**Published:** 2014-09-29

**Authors:** Martin Andersson, Lennart Johansson, David Minarik, Sigrid Leide-Svegborn, Sören Mattsson

**Affiliations:** 1 Medical Radiation Physics, Department of Clinical Sciences Malmö, Lund University, Skåne University Hospital, Malmö, Sweden; 2 Department of Radiation Sciences, Umeå University, Umeå, Sweden

**Keywords:** Radiopharmaceuticals, Internal dosimetry, Diagnostics, Nuclear medicine, ICRP

## Abstract

**Background:**

Effective dose represents the potential risk to a population of stochastic effects of ionizing radiation (mainly lethal cancer). In recent years, there have been a number of revisions and updates influencing the way to estimate the effective dose. The aim of this work was to recalculate the effective dose values for the 338 different radiopharmaceuticals previously published by the International Commission on Radiological Protection (ICRP).

**Method:**

The new estimations are based on information on the cumulated activities per unit administered activity in various organs and tissues and for the various radiopharmaceuticals obtained from the ICRP publications 53, 80 and 106. The effective dose for adults was calculated using the new ICRP/International Commission on Radiation Units (ICRU) reference voxel phantoms and decay data from the ICRP publication 107. The ICRP human alimentary tract model has also been applied at the recalculations. The effective dose was calculated using the new tissue weighting factors from ICRP publications 103 and the prior factors from ICRP publication 60. The results of the new calculations were compared with the effective dose values published by the ICRP, which were generated with the Medical Internal Radiation Dose (MIRD) adult phantom and the tissue weighting factors from ICRP publication 60.

**Results:**

For 79% of the radiopharmaceuticals, the new calculations gave a lower effective dose per unit administered activity than earlier estimated. As a mean for all radiopharmaceuticals, the effective dose was 25% lower. The use of the new adult computational voxel phantoms has a larger impact on the change of effective doses than the change to new tissue weighting factors.

**Conclusion:**

The use of the new computational voxel phantoms and the new weighting factors has generated new effective dose estimations. These are supposed to result in more realistic estimations of the radiation risk to a population undergoing nuclear medicine investigations than hitherto available values.

**Electronic supplementary material:**

The online version of this article (doi:10.1186/2197-7364-1-9) contains supplementary material, which is available to authorized users.

## Background

The sum of the radiation-risk weighted equivalent dose to organs and tissues in the human body (the effective dose) represents the potential risk from stochastic effects (mainly lethal cancer) of radiation. Thus, it makes it possible to compare various procedures involving ionizing radiation for radiation protection purposes. The effective dose is primarily intended as an important parameter for the planning and optimization of radiation protection and not as a quantity for individual risk estimates, as patient-specific parameters may vary significantly from the assumptions made in the risk models [1]. Moreover, the effective dose cannot be applied for therapy with radiopharmaceuticals as it only considers the stochastic effects.

The effective dose is based upon risk data used to obtain the sex-averaged tissue weighting factors. The idea was first introduced by the International Commission on Radiological Protection (ICRP) in 1977 [2], and later, at the Stockholm meeting [3], the ICRP assigned the term ‘effective dose equivalent’ and the symbol ‘H_E_’ to this new concept. Up to now, the weighting factors have been revised twice and the name of the quantity changed to effective dose (E) [1, 4]. The absorbed doses to organs and tissues and the effective dose per unit administered activity for radiopharmaceuticals found in the ICRP publications 53, 80 and 106, are all calculated based on biokinetic data from these publications and using the mathematical Medical Internal Radiation Dose (MIRD) phantoms from Cristy and Eckerman [5]. The adult male and adult female ICRP/International Commission on Radiation Units (ICRU) computational voxel phantoms were in 2007 approved by ICRP and adopted by ICRU in 2008 as reference phantoms for dosimetric calculations [6]. These phantoms were constructed by adjusting the voxel phantoms Golem [7] and Laura [8] to the organ masses given in the ICRP publication 89 [9]. Unlike for the previous phantoms, specific-absorbed fractions (SAF values) for electrons are now also simulated using Monte Carlo methods and published by Zankl et al*.* [10].

In the present study, the absorbed dose is calculated for males and females separately using the new phantoms, and the effective dose is then obtained by applying the organ-specific weighting factors to the arithmetic mean of the male and female dose equivalent [1]. For calculating the absorbed dose to organ and tissues as well as the effective dose, a computer program was developed [11]. The program includes the new adult phantoms and the present ICRP assumptions and definitions.

The previously used mathematically describable MIRD-phantoms were developed using highly simplified organ shapes, which sometimes resulted in less realistic distances within and between organs. For a limited number of radiopharmaceuticals, and for adults, it has been shown that there is a difference between earlier estimations of the effective dose and the results of the calculations using the new ICRP/ICRU reference phantoms and the new ICRP tissue weighting factors [10–12]. The aim of this work was to use published biokinetic data [13–15] as a base for a complete recalculation of the effective dose for all radiopharmaceuticals hitherto published by the ICRP, using the new adult reference phantoms [6] and the ICRP publication 103 tissue weighting factors [1].

## Method

### Absorbed dose and effective dose

The mean absorbed dose to a target region (*r*_*T*_) is calculated by [16]

1 $$D\left(r_{T},T_{D}\right)=\sum_{r_{s}}\tilde{A}\left(r_{s},T_{D}\right)S\left(r_{T}\leftarrow r_{s}\right)\;\left[\mathrm{Gy}\right]$$

where *Ã*(*r*_*s*_*,T*_*D*_) is the time-integrated activity, i.e. the total number of disintegrations, in source region *r*_*S*_ from intake to the time *T*_*D*_, and *S*(*r*_*T*_ *← r*_*S*_) is the mean absorbed dose in target region *r*_*T*_ per nuclear transformation in source region *r*_*S*_.

The total number of disintegrations is calculated by $\tilde{A}\left(r_{s},T_{D}\right)=\int_{0}^{T_{D}}A\left(r_{s},t\right)\mathrm{dt}$ where *A(r*_*S*_*,t)* is the activity of the radiopharmaceutical in source region *r*_*S*_ at time *t*. The *S*(*r*_*T*_ ← *r*_*S*_) is generated with radionuclide decay scheme and Monte Carlo simulated absorbed fractions

2 $$S\left(r_{T}\leftarrow r_{S}\right)=\sum_{i}\Delta \Phi \left(r_{T}\leftarrow r_{S},E_{i}\right)\;\left[\mathrm{Gy}/\mathrm{Bq}\right]$$

where ∆_*i*_ *= E*_*i*_*Y*_*i*_ and Φ(*r*_*T*_ *← r*_*S*_*,E*_*i*_) = φ_*i*_(*r*_*r*_ *← r*_*S*_*,E*_*i*_)/*m*(*r*_*T*_)_ is the mass of the target organ *T*, φ_*i*_ is the absorbed fraction, *Y*_*i*_ is the yield and *E*_*i*_ is the mean energy of the *i* th nuclear transition of the radionuclide. The *S*(*r*_*T*_ *← r*_*S*_) is in units of gray per becquerel if *M*(*r*_*T*_) is in kilograms and *E* is in Joules.

To estimate the risk for radiation-induced cancer and heritable diseases for a general population, the mean absorbed dose to the total body is insufficient information. In order to correlate stochastic effects and ionizing radiation, two types of weighting factors are used to calculate the effective dose:

3 $$E=\sum_{T}w_{T}\sum_{R}w_{R}D_{R}\left(r_{T},T_{D}\right)\;\left[\mathrm{Sv}\right]$$

where *D*_*R*_(*r*_*T*_,*T*_*D*_) is the mean absorbed dose, *w*_*R*_ is the radiation weighting factor of radiation type *R*, and *w*_T_ is the tissue weighting factor assigned by the ICRP to the different organs and tissues representing the relative detrimental effects [1]. For all radiation types used in diagnostic medical exposure, *w*_*R*_ is 1.

### From MIRD adult phantom to ICRP/ICRU adult reference male and female phantoms

The adult ICRP/ICRU reference computational phantoms for male and female include 63 source organs and 73 target organs [6]. For every source-target combination, the specific absorbed fractions have been calculated for electrons ranging from 10 keV to 10 MeV [10]. The MIRD adult phantoms include 25 source organs and 25 target organs [5], and 12 SAF values ranging from 10 keV to 4 MeV have been calculated for mono-energetic photons only. For the stylized phantom the biokinetic model describing the gastrointestinal tract is presented in ICRP publication 30 [17]. It was built up from the four regions: stomach, small intestine, upper large intestine and lower large intestine. In the new voxel phantom, which is designed to agree with the human alimentary tract model described in ICRP publication 100 [18], the gastrointestinal tract is now segmented as oral cavity, oesophagus, stomach, small intestine, right colon, left colon and rectosigmoid colon [9, 19].

### Assumptions in the estimation of the effective dose

All calculations were made with the decay properties (energies and yields) tabulated in the ICRP publication 107 database [20]. For the photons, a cutoff SAF value was introduced for large distances between source and target regions and low initial energies. For decay energies less than the cutoff energy for the simulations, a restrictive approach was used by applying the corresponding cutoff SAF value.

In the biokinetic model, the urinary bladder filling and emptying follows the ICRP standardized voiding interval [15] to calculate the time-integrated activity in the urinary bladder content. For the calculation of the absorbed dose to the urinary bladder wall as well as to other organs and tissues, the urinary content is assumed to have a constant volume of 200 ml [5].

To calculate the absorbed dose to radiosensitive organs and tissues from the data for ‘Cumulated activity in organ or tissue S per unit administered activity’ published by ICRP in publication 53, 80 or 106 [13–15] for different radiopharmaceuticals, some further adjustments were made:

### Gastrointestinal system

For all radiopharmaceuticals that are excreted through the gastrointestinal system, calculations are made applying the new ICRP human alimentary tract model [18] to estimate the total number of disintegrations in the new regions of the gastrointestinal tract.

### Bone

For bone-seeking radiopharmaceuticals or radionuclides, for which the distribution of cumulated activity between the cortical and trabecular bone is unknown, the assumption is that substances with an effective half-time shorter than 15 days are surface-deposited; otherwise, they are distributed uniformly throughout the entire volume of trabecular and cortical bone [13].

### Other organs and tissue

For the source region defined as ‘other organs and tissues,’ the dose calculations are performed applying a method using a formally exact solution, derived by Roedler and Kaul [21]. The value is generated by adjusting the source regions ‘total body’ by removing the contribution of the source regions already accounted for and calculated as

4 $$S\left(r_{T}\leftarrow r_{\mathrm{Other}\;\mathrm{organs}\;\mathrm{and}\;\mathrm{tissues}}\right)=\frac{m_{\mathrm{TB}}S\left(r_{T}\leftarrow r_{\mathrm{TB}}\right)-\sum_{S}m_{S}S\left(r_{T}\leftarrow r_{S}\right)}{m_{\mathrm{TB}}-\sum_{S}m_{S}}$$

where *S*(*r*_*T*_ *← r*_*B*_) is the dose conversion factor from the source region ‘total body’ to the target region *r*_*T*_. *m*_TB_ and *m*_*S*_ are the masses of the total body and the source region *S*, respectively and *S*(*r*_*T*_ *← r*_*S*_) is the dose conversion factor from one source organ *S* to the target region *T*.

### Blood

Radiopharmaceuticals, which to a significant extent are present in circulating blood, were in ICRP publication 53 [13] assumed to be distributed by the fractional blood volume. For ICRP publication 80 and 106, the circulating blood was described using Leggett and Williams' blood circulation model [22]. For calculations with the new phantoms, the reference values for blood content given in ICRP publication 89 were used to distribute the activity in the circulating blood [9]. In a few cases, a substitute region was used when the different source regions were inconsistent, e.g. heart content was used as a substitute for the aorta.

### Walls of the colon

In the case where radionuclides were deposited in the walls of the colon, the distribution to the activity in the walls was recalculated from the ‘old gastrointestinal tract regions’ to the regions described in the ICRP Human alimentary tract model [18]. The time-integrated activity in the upper large intestine and the lower large intestine was converted to the right colon, left colon and rectosigmoid colon by a conversion factor based on the masses of the different regions [9, 19]

5 $$\tilde{A}\left(r_{\mathrm{Right}\;\mathrm{colon}},T_{D}\right)=0.71*\tilde{A}\left(r_{\mathrm{Upper}\;\text{large}\;\mathrm{int}\mathrm{estine}},T_{D}\right)$$

6 $$\begin{array}{ll} \tilde{A}\left(r_{\mathrm{Left}\;\mathrm{colon}},T_{D}\right)= & 0.29*\tilde{A}\left(r_{\mathrm{Upper}\;\mathrm{large}\;\mathrm{intentine}},T_{D}\right)+0.56\\ {}*\tilde{A}\left(r_{\mathrm{Lower}\;\mathrm{large}\;\mathrm{intentine}},T_{D}\right) \end{array}$$

7 A˜rRectosigmoidcolon,TD=0.44*A˜rLowerlargeintestine,TD

where *Ã*(*r*_Upper large intestine_,*T*_*D*_) and *Ã*(*r*_Lower large intestine_,*T*_*D*_) is the time integrated activity in the upper large intestine wall and lower large intestine wall respectively and *Ã*(*r*_Rectosigmoid colon_,*T*_*D*_), *Ã*(*r*_Left colon_,*T*_*D*_) and *Ã*(*r*_Right colon_,*T*_*D*_) are the total number of disintegrations in the new regions.

### ICRP tissue weighting factors in Publication 60 versus those in Publication 103

One major difference between the ICRP publication 60 and 103 is that the tissue weighting factor for the remainder is now equally divided between 13 specified organs for males and females respectively [1]. When calculating the effective dose according to the ICRP 60 system, the weighting factor for the remainder was applied to a mass weighted absorbed dose to a number of specified remaining organs, there was also a splitting rule that stated that the half weighting factor (0.25) should be applied to a single remaining organ, if this organ receives the highest absorbed dose of all organs.

### Effective dose comparison

The organ and tissue equivalent dose values obtained with the voxel phantom were used to determine the effective doses based on the tissue weighting factors from ICRP publication 60 as well as those from publication 103. To calculate the dose to the colon, the same assumption was used as earlier mentioned to convert from the new intestine regions to the older ones. The equivalent doses for the Reference Male and the Reference Female are multiplied with the ICRP publication 103 tissue weighting factors and then averaged to estimate the effective dose for a Reference Person [1]. Calculations were also performed for each gender separately. The ICRP publication 60 tissue weighting factors were all applied to organ-absorbed doses averaged between males and females in order to obtain the effective dose.

Calculations were performed for each radiopharmaceutical in two different ways, either (a) the effective dose was calculated using the new voxel phantom with weighting factors from ICRP publication 60 [4] or (b) the calculations were made using the new phantoms and the new ICRP publication 103 tissue weighting factors [1]. Some of the radiopharmaceuticals published in ICRP publication 53 [13] are included in the recalculation from effective dose equivalent [2] to effective dose in ICRP publication 80 [14] or have been completely modified in the later ICRP publication 106 [15]. The others were calculated using the absorbed doses in ICRP publication 53 to get the effective dose. In some cases in the ICRP publication 53 [13], two different biokinetic models are presented, one describing the biokinetics in the whole body and one organ-specific model. If so, the time-integrated activities for the specific organs are chosen and their contribution is subtracted from the ‘total body’. The remaining activities are used as the source region ‘other organs and tissues’.

## Results

**Figure 1:**
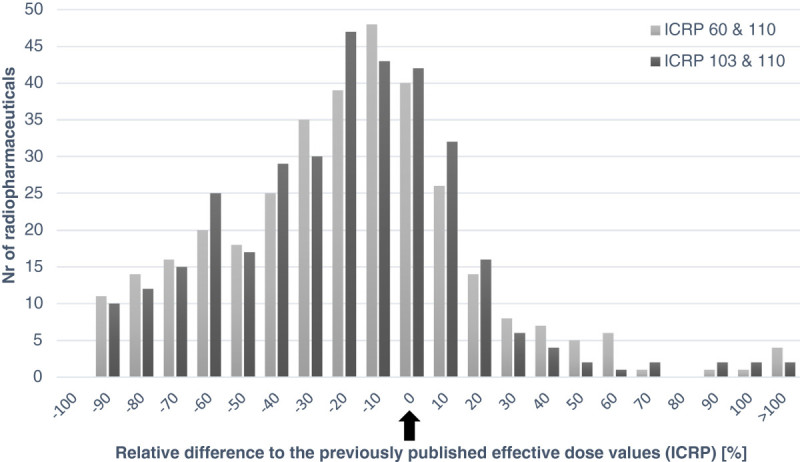
**A histogram of the relative difference between different dose values.** The relative difference between the old published effective dose per unit administered activity and the effective dose values calculated with the new phantom (ICRP 110) and with (1) the new (ICRP 103) and (2) the previous (ICRP 60) tissue weighting factors. The arrow indicates identical results between old and new estimations.

New values of effective dose per unit administered activity (E/A_0_) for adults and for the 55 different radiopharmaceuticals included in ICRP publication 106 are presented in Table 1. The new values for all the 338 radiopharmaceuticals are available as a supplement to the present paper (Additional file 1: Table S1). The calculated values are lower than earlier presented values for 79% of the radiopharmaceuticals. As a mean for all 338 radiopharmaceuticals, the values are 25% lower. The observed reduction depends to a larger degree on the use of the new adult computational voxel phantoms than on the change to new tissue weighting factors. The effective doses are larger for females than for males in 62% of all 338 radiopharmaceuticals. The black bars in Figure 1 represent the distribution of the percentage difference between the new and the old effective dose for all radiopharmaceuticals. The grey bars show the differences between the effective doses calculated with the new phantoms and the previous phantom using the previous tissue weighting factors. Only for ^125^I Iodine Hippuran with unilateral renal blockage and an abnormal kidney function there is a difference of more than 100% between the new and the old E/A_0_ values.

**Table 1:** **Effective dose from the 55 radiopharmaceuticals in ICRP publication 106, determined using three different methods**

	(E/A _0_)1 [mSv/MBq]	(E/A _0_)2 [mSv/MBq]	((E/A _0_)2 − (E/A _0_)1)/(E/A _0_)1 [%]	(E/A _0_)3 [mSv/MBq]	((E/A _0_)3 − (E/A _0_)1)/(E/A _0_)1[%]	(E/A _0_)3 male [mSv/MBq]	(E/A _0_)3 female [mSv/MBq]
Phantom	MIRD	ICRP/ICRU		ICRP/ICRU		ICRP/ICRU	ICRP/ICRU
w _T_	ICRP 60	ICRP 60		ICRP 103		ICRP 103	ICRP 103
Radiopharmaceuticals							
^3^H Tritium-labelled neutral fat & free fatty acids	2.2E-01	9.34E-02	−58	1.72E-01	−22	2.38E-01	1.05E-01
^11^C Carbon acetate	3.5E-03	4.37E-03	25	4.20E-03	20	4.08E-03	4.31E-03
^11^C Carbon amino acids	5.6E-03	4.43E-03	−21	4.62E-03	−18	4.89E-03	4.34E-03
^11^C Carbon brain receptor substances	4.3E-03	3.22E-03	−25	3.56E-03	−17	3.69E-03	3.42E-03
^11^C Carbon methionine	8.4E-03	5.39E-03	−36	5.49E-03	−35	5.69E-03	5.28E-03
^11^C Carbon (2-^11^C)thymidine	2.7E-03	2.36E-03	−13	2.53E-03	−6	2.61E-03	2.45E-03
^11^C Carbon, realistic maximum	1.1E-02	4.99E-03	−55	5.46E-03	−50	6.12E-03	4.79E-03
^14^C Carbon-labelled neutral fat and free fatty acids	2.1E + 00	1.75E + 00	−17	2.75E + 00	31	3.37E + 00	2.16E + 00
^14^C Carbon-labelled urea, normal case, orally administered	3.1E-02	2.32E-02	−25	2.65E-02	−15	2.64E-02	2.66E-02
^15^O Oxygen water	1.1E-03	9.07E-04	−18	8.29E-04	−25	8.30E-04	8.29E-04
^18^F Fluoride-labelled amino acids	2.3E-02	1.75E-02	−24	1.86E-02	−19	1.97E-02	1.74E-02
^18^F Fluoride-labelled brain receptor substances	2.8E-02	1.89E-02	−33	1.91E-02	−32	1.93E-02	1.89E-02
^18^F Fluoride FDG	1.9E-02	1.50E-02	−21	1.59E-02	−16	1.66E-02	1.53E-02
^18^F Fluoride l-dopa	2.5E-02	1.51E-02	−40	1.68E-02	−33	1.85E-02	1.52E-02
^51^Cr Chromium EDTA	2.0E-03	1.39E-03	−31	1.56E-03	−22	1.76E-03	1.36E-03
^67^Ga Gallium citrate	1.0E-01	7.66E-02	−23	8.59E-02	−14	8.58E-02	8.59E-02
^68^Ga Gallium-labelled EDTA	4.0E-02	2.35E-02	−41	2.37E-02	−41	2.45E-02	2.29E-02
^75^Se Selenium-labelled amino acids	2.2E + 00	2.03E + 00	−8	2.21E + 00	0	2.33E + 00	2.09E + 00
^75^Se Selenium-labelled bile acid SeHCAT	6.9E-01	2.37E-01	−66	2.77E-01	−60	2.76E-01	2.77E-01
^99m^Tc Technetium apcitide	4.7E-03	1.90E-03	−60	2.05E-03	−56	2.01E-03	2.09E-03
^99m^Tc Technetium-labelled small colloids, intratumoural adm. time to removal 18 h	2.0E-03	3.14E-03	57	3.96E-03	98	3.49E-03	4.43E-03
^99m^Tc Technetium-labelled small colloids, intratumoural adm time to removal 6 h	1.2E-03	1.78E-03	48	2.24E-03	87	1.98E-03	2.50E-03
^99m^Tc Technetium EC, normal renal function	6.3E-03	3.69E-03	−41	4.23E-03	−33	5.12E-03	3.33E-03
^99m^Tc Technetium ECD	7.7E-03	5.36E-03	−30	5.75E-03	−25	6.13E-03	5.36E-03
^99m^Tc Technetium furifosmin, exercise	8.9E-03	6.25E-03	−30	6.67E-03	−25	6.73E-03	6.60E-03
^99m^Tc Technetium furifosmin, resting subject	1.0E-02	6.53E-03	−35	6.99E-03	−30	7.07E-03	6.91E-03
^99m^Tc Technetium-labelled HIG	7.0E-03	4.72E-03	−33	4.59E-03	−34	4.89E-03	4.29E-03
^99m^Tc Technetium-labelled HM-PAO	9.3E-03	1.06E-02	14	1.01E-02	9	9.93E-03	1.04E-02
Tc-99 m Technetium-labelled IDA derivatives, normal hepato-biliary conditions	1.7E-02	7.70E-03	−55	8.62E-03	−49	8.58E-03	8.66E-03
^99m^Tc Technetium-labelled MAA	1.1E-02	1.29E-02	17	1.02E-02	−7	9.54E-03	1.08E-02
^99m^Tc Technetium-labelled MAG3, normal renal function	7.0E-03	4.05E-03	−42	4.65E-03	−34	5.68E-03	3.62E-03
^99m^Tc Technetium-labelled non-absorbable markers, orally administered fluids	1.9E-02	9.88E-03	−48	1.06E-02	−44	1.04E-02	1.08E-02
^99m^Tc Technetium-labelled non-absorbable markers, orally administered solids	2.4E-02	1.08E-02	−55	1.14E-02	−53	1.11E-02	1.18E-02
^99m^Tc Technetium-labelled MIBI, exercise	9.0E-03	6.06E-03	−33	6.55E-03	−27	6.57E-03	6.52E-03
^99m^Tc Technetium-labelled MIBI, resting subject	7.9E-03	6.58E-03	−17	7.03E-03	−11	6.95E-03	7.11E-03
^99m^Tc Technetium-labelled monoclonal antibodies, intact antibody	1.2E-02	8.27E-03	−31	8.18E-03	−32	7.95E-03	8.40E-03
^99m^Tc Technetium pertechnegas	1.2E-02	1.46E-02	22	1.46E-02	22	1.41E-02	1.50E-02
^99m^Tc Technetium pertechnetate, intravenous blocking agent given	4.2E-03	3.66E-03	−13	4.12E-03	−2	4.47E-03	3.78E-03
^99m^Tc Technetium pertechnetate, intravenous no blocking agent given	1.3E-02	1.55E-02	19	1.59E-02	22	1.55E-02	1.64E-02
^99m^Tc Technetium pertechnetate orally, no blocking agent	1.4E-02	6.02E-03	−57	6.38E-03	−54	6.33E-03	6.43E-03
^99m^Tc Technetium-labelled phosphates and phosphonates, normal uptake and excretion	5.8E-03	3.80E-03	−34	4.31E-03	−26	4.86E-03	3.75E-03
^99m^Tc Technetium-labelled erythrocytes	7.0E-03	2.57E-03	−63	2.69E-03	−62	2.67E-03	2.71E-03
^99m^Tc Technetium technegas	1.5E-02	1.87E-02	25	1.36E-02	−9	1.24E-02	1.49E-02
^99m^Tc Technetium tetrofosmin, exercise	6.9E-03	5.18E-03	−25	5.76E-03	−17	5.86E-03	5.66E-03
^99m^Tc Technetium tetrofosmin, resting subject	8.0E-03	5.84E-03	−27	6.29E-03	−21	6.36E-03	6.22E-03
^99m^Tc Technetium-labelled white blood cells (leukocytes)	1.1E-02	9.60E-03	−13	7.17E-03	−35	6.81E-03	7.54E-03
^111^In Indium-labelled HIG	1.7E-01	1.39E-01	−18	1.41E-01	−17	1.44E-01	1.38E-01
^111^In Indium-labelled monoclonal antibodies, intact antibody	3.3E-01	2.14E-01	−35	2.24E-01	−32	2.17E-01	2.32E-01
^111^In Indium octreotide	5.4E-02	8.02E-02	49	6.87E-02	27	6.79E-02	6.96E-02
^123^I Iodide, thyroid uptake 35%	2.2E-01	2.72E-01	24	2.33E-01	6	2.12E-01	2.53E-01
^123^I Iodine BMIPP	1.6E-02	1.37E-02	−14	1.57E-02	−2	1.62E-02	1.52E-02
^123^I Iodine IPPA	1.6E-02	1.38E-02	−14	1.58E-02	−1	1.63E-02	1.53E-02
^123^I Iodine-labelled brain receptor substances	5.0E-02	3.33E-02	−33	3.30E-02	−34	3.18E-02	3.43E-02
^123^I Iodine Hippuran, normal renal function	1.2E-02	7.41E-03	−38	8.32E-03	−31	1.00E-02	6.62E-03
^123^I Iodine MIBG	1.3E-02	1.14E-02	−12	1.32E-02	2	1.36E-02	1.27E-02
^123^I Iodine-labelled monoclonal antibodies, intact antibody	2.9E-02	2.33E-02	−20	2.18E-02	−25	1.11E-03	1.24E-03
^124^I Iodide, thyroid uptake 35%	1.5E + 01	1.51E + 01	1	1.28E + 01	−15	1.14E + 01	1.41E + 01
^125^I Iodide, thyroid uptake 35%	1.4E + 01	1.98E + 01	41	1.66E + 01	19	1.50E + 01	1.83E + 01
^131^I Iodide, thyroid uptake 35%	2.4E + 01	2.72E + 01	13	2.22E + 01	−8	2.03E + 01	2.41E + 01
^131^I Iodine, Hippuran, normal renal function	5.2E-02	1.65E-02	−68	1.80E-02	−65	2.10E-02	1.51E-02
^131^I Iodine-labelled monoclonal antibodies, intact antibody	4.7E-01	3.13E-01	−33	2.57E-01	−45	2.49E-01	2.66E-01
^131^I Iodine NP59	1.8E + 00	1.94E + 00	8	1.73E + 00	−4	1.62E + 00	1.84E + 00
^201^Tl Thallium ion	1.4E-01	1.21E-01	−14	*1.02E-01*	−27	1.07E-01	9.76E-02

(E/A_0_)1 is the previously published effective dose per unit administered activity (E/A_0_) by ICRP, (E/A_0_)2 is (E/A_0_) dose calculated with the new phantoms and old tissue weighting factors while (E/A_0_)3 is with the new phantoms and new weighting factors. (E/A_0_)2 − (E/A_0_)1))/(E/A_0_)1 and ((E/A_0_)3 − (E/A_0_)1)/(E/A_0_)1 is the difference in percentage (%) of the new values compared to the old. (E/A_0_)3 male and (E/A_0_)3 female are the estimations generated from the equivalent dose of each gender separately using the new phantoms and new weighting factors.

## Discussion

The effective dose has been calculated using the new computational phantom, recent radionuclide decay data, the new human alimentary tract model and the tissue weighting factors given in ICRP publication 103. How these new data and calculation assumptions affect the effective dose depends on both the source regions included in the biokinetic model and the physical decay for each radiopharmaceutical. Hadid et al. [12] have investigated in detail what the main differences are between the old and the new phantoms with respect to the effective dose, and they have also calculated the absorbed and effective dose for 15 commonly used radiopharmaceuticals. The two major factors influencing the calculation results of the absorbed dose to the target regions are the improved data on absorbed fractions for electrons, especially for walled organs, and the use of a realistic voxel phantom instead of the stylized phantom used earlier [12]. Both of these factors cause a reduction in the estimations of the effective dose. Figure 1 shows that changing the phantoms has a larger impact on the effective dose than the new tissue weighting factors. The effective dose per unit administered activity is on average larger for women than for men. The main difference between the effective doses for women and men occurs for radiopharmaceuticals administered orally. For radiopharmaceuticals with a significant uptake in adipose tissue as for ^14^C- and ^3^H-labelled neutral fat and free fatty acids or in the male gonads, the effective dose will be higher for males than for females. It should also be noted that the differences in the effective dose between genders is due to the phantoms. The stochastic effects for a specific radiosensitive organ can vary between genders. However, the tissue weighting factors are published as sex-averaged and the biokinetic models are also non-gender specific, except for the 4 h longer female transit time in the colon. There are also some other general observations. As earlier shown [23] for intravenous-administered radiopharmaceuticals labelled with a radionuclide of short physical half-life, the variation of E/A_0_ is limited. For ^18^F-labelled substances, E/A_0_ varies between 0.013 and 0.019 mSv/MBq (less than a factor of 1.5). For ^11^C-substances, E/A_0_ varies between 0.0025 and 0.0055 mSv/MBq (around a factor of 2.2). Also for ^99m^Tc-labelled substances, the range of E/A_0_ values is limited to 0.0017 to 0.016 mSv/MBq (a factor of 9.6). For radiopharmaceuticals where the radionuclide has a longer physical half-life, the differences between various substances are larger and more dependent on the biokinetic behaviour of the substances. For all the ^18^F substances, there is a reduction in effective dose estimation by 29% in average. For ^11^C-substances, two radiopharmaceuticals show a higher effective dose and 11 have a lower effective dose than previously published values. In 50 of the 62 ^99m^Tc-substances, the effective dose estimations give lower values than previous estimations.

In Sweden, the collective effective dose from diagnostic examinations in nuclear medicine was estimated to 334 manSv in 2012 using the old effective dose estimations. Using the new estimations, the collective effective dose is estimated at 292 manSv, i.e. 13% lower value than earlier estimated.

## Conclusions

This study shows that the introduction of more realistic gender-specific voxel phantoms will lead to a reduction of the estimated effective dose for a majority of radiopharmaceuticals. The impact of the new phantom, improved calculation methods and tissue weighting factors is still within a factor of two of the former values for almost all radiopharmaceuticals.

For 268 radiopharmaceuticals out of 338, the new calculations show lower effective dose values than previous estimates. For 68 radiopharmaceuticals, the new calculations results in an increased value of the estimated effective dose. Therefore, hospitals, referring physicians, research groups and ethical committees should be encouraged to use the updated versions of the effective dose estimations to be in line with the current dosimetric methods and radiation risk estimations.

## Electronic supplementary material

Additional file 1: Table S1: Effective dose from all the radiopharmaceuticals published by the ICRP, determined using three different methods. (E/A_0_)1 is the previously published effective dose per unit administered activity (E/A_0_) by ICRP, (E/A_0_)2 is (E/A_0_) dose calculated with the new phantoms and old tissue weighting factors while (E/A_0_)3 is with the new phantoms and new weighting factors. (E/A_0_)2 − (E/A_0_)1))/(E/A_0_)1 and ((E/A_0_)3 − (E/A_0_)1)/(E/A_0_)1 is the difference in% of the new values compared to the old. (E/A_0_)3 male and (E/A_0_)3 are the estimations generated from the equivalent dose of each gender separately using the new phantoms and new weighting factors. (DOC 466 KB)

Below are the links to the authors’ original submitted files for images.

Authors’ original file for figure 1
