# Erratum to: Effective dose to adult patients from 338 radiopharmaceuticals estimated using ICRP biokinetic data, ICRP/ICRU computational reference phantoms and ICRP 2007 tissue weighting factors

**DOI:** 10.1186/s40658-015-0121-4

**Published:** 2015-09-30

**Authors:** Martin Andersson

**Affiliations:** Medical Radiation Physics, Department of Clinical Sciences Malmö, Lund University, Skåne University Hospital, Malmö, Sweden; Department of Radiation Sciences, Umeå University, Umeå, Sweden

Correction of the article

In the results section of the abstract the two sentences

1. For 79 % of the radiopharmaceuticals, the new calculations gave a lower effective dose per unit administered activity than earlier estimated.” Should be “For 63 % of the radiopharmaceuticals, the new calculations gave a lower effective dose per unit administered activity than earlier estimated.”
2. “As a mean for all radiopharmaceuticals, the effective dose was 25 % lower.” Should be“As a mean for all radiopharmaceuticals, the effective dose was 11 % lower.”

In the results section in the main article four sentences should be changed

1. “The calculated values are lower than earlier presented values for 79 % of the radiopharmaceuticals.” Should be “The calculated values are lower than earlier presented values for 63 % of the radiopharmaceuticals.”
2. “As a mean for all 338 radiopharmaceuticals, the values are 25 % lower.” Should be “As a mean for all 338 radiopharmaceuticals, the values are 11 % lower.”
3. “The effective doses are larger for females than for males in 62 % of all 338 radiopharmaceuticals.” Should be “The effective doses are larger for females than for males in 99 % of all 338 radiopharmaceuticals.”
4. “Only for ^125^I Iodine Hippuran with unilateral renal blockage and an abnormal kidney function there is a difference of more than 100 % between the new and the old E/A_0_ values.” Should be “Only for ^99m^Tc Apcitide and ^99m^Tc labelled colloids, small colloids and normal liver condition there is a difference of more than 100 % between the new and the old E/A_0_ values.”

In the Discussion section in the main article eight sentences should be changed

1. “For radiopharmaceuticals with a significant uptake in adipose tissue as for ^14^C- and ^3^H-labelled neutral fat and free fatty acids or in the male gonads, the effective dose will be higher for males than for females.” Should be “For radiopharmaceuticals with a significant uptake in adipose tissue as for ^14^C- and ^3^H-labelled neutral fat and free fatty acids, the effective dose will be higher for males than for females.”
2. “For ^18^ F-labelled substances, E/A_0_ varies between 0.013 and 0.019 mSv/MBq (less than a factor of 1.5).” Should be “For ^18^ F-labelled substances, E/A_0_ varies between 0.013 and 0.021 mSv/MBq (a factor of 1.6).”
3. “For ^11^C-substances, E/A_0_ varies between 0.0025 and 0.0055 mSv/MBq (around a factor of 2.2).” Should be “For ^11^C-substances, E/A_0_ varies between 0.0011 and 0.0087 mSv/MBq (around a factor of 8.0). “
4. “Also for ^99m^Tc-labelled substances, the range of E/A_0_ values is limited to 0.0017 to 0.016 mSv/MBq (a factor of 9.6).” Should be “Also for ^99m^Tc-labelled substances, the range of E/A_0_ values is limited to 0.0022 to 0.020 mSv/MBq (a factor of 8.8).”
5. “For all the ^18^ F substances, there is a reduction in effective dose estimation by 29 % in average.” Should be “For all the ^18^ F substances, there is a reduction in effective dose estimation by 26 % in average.”
6. “For ^11^C-substances, two radiopharmaceuticals show a higher effective dose and 11 have a lower effective dose than previously published values.” Should be “For ^11^C-substances, nine radiopharmaceuticals show a higher effective dose and four have a lower effective dose than previously published values.”
7. “In 50 of the 62 ^99m^Tc-substances, the effective dose estimations give lower values than previous estimations.” Should be “In 38 of the 62 ^99m^Tc-substances, the effective dose estimations give lower values than previous estimations.”
8. “Using the new estimations, the collective effective dose is estimated at 292 manSv, i.e. 13 % lower value than earlier estimated.” Should be “Using the new estimations, the collective effective dose is estimated at 295 manSv, i.e. 12 % lower value than earlier estimated.”

In the Conclusions there are two sentences that should be changed

1. “For 268 radiopharmaceuticals out of 338, the new calculations show lower effective dose values than previous estimates.” Should be “For 212 radiopharmaceuticals out of 338, the new calculations show lower effective dose values than previous estimates.”
2. “For 68 radiopharmaceuticals, the new calculations results in an increased value of the estimated effective dose.” Should be “For 120 radiopharmaceuticals, the new calculations results in an increased value of the estimated effective dose.”

Figure 1 should be changed to (only the figure not the text)

**Fig. 1:**
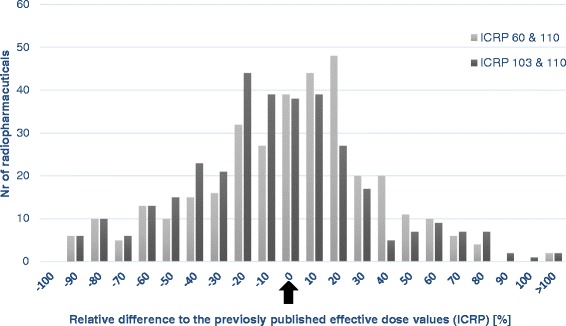
A histogram of the relative difference between different dose values. The relative difference between the old published effective dose per unit administered activity and the effective dose values calculated with the new phantom (ICRP 110) and with (1) the new (ICRP 103) and (2) the previous (ICRP 60) tissue weighting factors. The arrow indicates identical results between old and new estimations

Almost all numbers have been changed in Table 1 and a new corrected Table 1 is presented below (table text to Table 1 does not need to be changed)

**Table 1:** Effective dose from the 55 radiopharmaceuticals in ICRP publication 106, determined using three different methods

Radiopharmaceuticals	(E/A_0_)1 [mSv/MBq]	(E/A_0_)2 [mSv/MBq]	((E/A_0_)2-(E/A_0_)1)/(E/A_0_)1 [%]	(E/A_0_)3 [mSv/MBq]	((E/A_0_)3-(E/A_0_)1)/(E/A_0_)1 [%]	(E/A_0_)3 male [mSv/MBq]	(E/A_0_)3 female [mSv/MBq]
Phantom	MIRD	ICRP/ICRU		ICRP/ICRU		ICRP/ICRU	ICRP/ICRU
w_T_	ICRP 60	ICRP 60		ICRP 103		ICRP 103	ICRP 103
^3^H Tritium labelled neutral fat & free fatty acids	2.2E-01	9.81E-02	−55	1.80E-01	−18	2.44E-01	1.16E-01
^11^C Carbon acetate	3.5E-03	4.26E-03	22	3.65E-03	4	3.33E-03	3.97E-03
^11^C Carbon amino acids	5.6E-03	5.76E-03	3	5.26E-03	−6	4.91E-03	5.61E-03
^11^C Carbon brain receptor substances	4.3E-03	3.70E-03	−14	3.62E-03	−16	3.23E-03	4.00E-03
^11^C Carbon methionine	8.4E-03	5.88E-03	−30	5.11E-03	−39	4.50E-03	5.72E-03
^11^C Carbon (2-^11^C)thymidine	2.7E-03	3.01E-03	11	3.04E-03	13	2.77E-03	3.32E-03
^11^C Carbon, realistic maximum	1.1E-02	6.04E-03	−45	5.08E-03	−54	4.36E-03	5.80E-03
^14^C Carbon labelled neutral fat & free fatty acids	2.1E + 00	1.88E + 00	−10	2.65E + 00	26	3.14E + 00	2.15E + 00
^14^C Carbon labelled urea, normal case, orally administered	3.1E-02	2.67E-02	−14	2.72E-02	−12	2.46E-02	2.98E-02
^15^O Oxygen water	1.1E-03	9.63E-04	−12	9.33E-04	−15	8.72E-04	9.93E-04
^18^ F Fluoride labelled amino acids	2.3E-02	2.27E-02	−1	2.07E-02	−10	1.92E-02	2.21E-02
^18^ F Fluoride labelled brain receptor substances	2.8E-02	2.01E-02	−28	2.02E-02	−28	1.82E-02	2.22E-02
^18^ F Fluoride FDG	1.9E-02	1.69E-02	−11	1.71E-02	−10	1.53E-02	1.88E-02
^18^ F Fluoride L-dopa	2.5E-02	1.75E-02	−30	1.57E-02	−37	1.37E-02	1.76E-02
^51^Cr Chromium EDTA	2.0E-03	1.65E-03	−18	1.43E-03	−29	1.23E-03	1.62E-03
^67^Ga Gallium citrate	1.0E-01	9.29E-02	−7	9.08E-02	−9	8.14E-02	1.00E-01
^68^Ga Gallium labelled EDTA	4.0E-02	2.41E-02	−40	2.14E-02	−47	1.89E-02	2.40E-02
^75^Se Selenium labelled amino acids	2.2E + 00	2.39E + 00	8	2.27E + 00	3	2.14E + 00	2.39E + 00
^75^Se Selenium labelled bile acid SeHCAT	6.9E-01	3.01E-01	−56	3.48E-01	−50	3.16E-01	3.79E-01
^99m^Tc Technetium apcitide	4.7E-03	1.13E-02	140	1.19E-02	153	1.10E-02	1.29E-02
^99m^Tc Technetium labelled small colloids, intratumoural adm. time to removal 18 h	2.0E-03	3.14E-03	57	3.96E-03	98	3.49E-03	4.43E-03
^99m^Tc Technetium labelled small colloids, intratumoural adm time to removal 6 h	1.2E-03	1.78E-03	48	2.24E-03	86	1.98E-03	2.50E-03
^99m^Tc Technetium EC, normal renal function	6.3E-03	4.63E-03	−27	3.66E-03	−42	3.04E-03	4.29E-03
^99m^Tc Technetium ECD	7.7E-03	5.97E-03	−23	5.64E-03	−27	5.01E-03	6.27E-03
^99m^Tc Technetium furifosmin, exercise	8.9E-03	6.57E-03	−26	6.78E-03	−24	6.16E-03	7.40E-03
^99m^Tc Technetium furifosmin, resting subject	1.0E-02	6.99E-03	−30	7.19E-03	−28	6.53E-03	7.85E-03
^99m^Tc Technetium labelled HIG	7.0E-03	9.83E-03	40	9.42E-03	35	8.93E-03	9.92E-03
^99m^Tc Technetium labelled HM-PAO	9.3E-03	1.05E-02	13	9.78E-03	5	8.95E-03	1.06E-02
Tc-99 m Technetium labelled IDA derivatives, normal hepato-biliary conditions	1.7E-02	9.39E-03	−45	9.73E-03	−43	8.93E-03	1.05E-02
^99m^Tc Technetium labelled MAA	1.1E-02	1.34E-02	22	1.40E-02	27	1.27E-02	1.53E-02
^99m^Tc Technetium labelled MAG3, normal renal function	7.0E-03	5.12E-03	−27	4.00E-03	−43	3.29E-03	4.70E-03
^99m^Tc Technetium labelled non-absorbable markers, orally administered fluids	1.9E-02	1.06E-02	−44	1.07E-02	−44	9.93E-03	1.14E-02
^99m^Tc Technetium labelled non-absorbable markers, orally administered solids	2.4E-02	1.13E-02	−53	1.15E-02	−52	1.07E-02	1.24E-02
^99m^Tc Technetium labelled MIBI, exercise	7.9E-03	6.55E-03	−17	6.29E-03	−20	5.80E-03	6.78E-03
^99m^Tc Technetium labelled MIBI, resting subject	9.0E-03	6.81E-03	−24	6.61E-03	−27	6.14E-03	7.07E-03
^99m^Tc Technetium labelled monoclonal antibodies, intact antibody	1.2E-02	1.17E-02	−3	1.08E-02	−10	9.95E-03	1.16E-02
^99m^Tc Technetium pertechnegas	1.2E-02	1.52E-02	26	1.50E-02	25	1.39E-02	1.61E-02
^99m^Tc Technetium pertechnetate, intravenous blocking agent given	4.2E-03	4.34E-03	3	4.02E-03	−4	3.58E-03	4.46E-03
^99m^Tc Technetium pertechnetate, intravenous no blocking agent given	1.3E-02	1.60E-02	23	1.58E-02	22	1.48E-02	1.68E-02
^99m^Tc Technetium pertechnetate orally, no blocking agent	1.4E-02	6.48E-03	−54	6.36E-03	−55	5.83E-03	6.89E-03
^99m^Tc Technetium labelled phosphates and phosphonates, normal uptake and excretion	5.7E-03	4.55E-03	−20	3.99E-03	−30	3.38E-03	4.59E-03
^99m^Tc Technetium labelled erythrocytes	7.0E-03	1.06E-02	51	1.11E-02	59	1.02E-02	1.20E-02
^99m^Tc Technetium technegas	1.5E-02	1.79E-02	19	1.90E-02	27	1.71E-02	2.08E-02
^99m^Tc Technetium tetrofosmin, exercise	6.9E-03	5.54E-03	−20	5.67E-03	−18	5.15E-03	6.20E-03
^99m^Tc Technetium tetrofosmin, resting subject	8.0E-03	5.92E-03	−26	6.15E-03	−23	5.57E-03	6.72E-03
^99m^Tc Technetium labelled white blood cells (leukocytes)	1.1E-02	1.28E-02	16	1.02E-02	−7	9.24E-03	1.12E-02
^111^In Indium labelled HIG	1.7E-01	2.23E-01	31	2.15E-01	26	1.99E-01	2.31E-01
^111^In Indium labelled monoclonal antibodies, intact antibody	3.3E-01	2.88E-01	−13	2.74E-01	−17	2.49E-01	2.99E-01
^111^In Indium octreotide	5.4E-02	6.74E-02	25	5.93E-02	10	5.34E-02	6.51E-02
^123^I Iodide, thyroid uptake 35 %	2.2E-01	2.59E-01	18	2.13E-01	−3	1.95E-01	2.30E-01
^123^I Iodine BMIPP	1.6E-02	1.70E-02	6	1.71E-02	7	1.56E-02	1.87E-02
^123^I Iodine IPPA	1.6E-02	1.72E-02	7	1.72E-02	8	1.56E-02	1.87E-02
^123^I Iodine labelled brain receptor substances	5.0E-02	3.60E-02	−28	3.65E-02	−27	3.30E-02	4.00E-02
^123^I Iodine Hippuran, normal renal function	1.2E-02	8.88E-03	−26	7.06E-03	−41	5.98E-03	8.15E-03
^123^I Iodine MIBG	1.3E-02	1.67E-02	28	1.67E-02	28	1.51E-02	1.82E-02
^123^I Iodine labelled monoclonal antibodies, intact antibody	2.9E-02	3.29E-02	13	2.94E-02	1	2.68E-02	3.21E-02
^124^I Iodide, thyroid uptake 35 %	1.5E + 01	1.41E + 01	−6	1.15E + 01	−23	1.05E + 01	1.25E + 01
^125^I Iodide, thyroid uptake 35 %	1.4E + 01	1.85E + 01	32	1.50E + 01	7	1.38E + 01	1.62E + 01
^131^I Iodide, thyroid uptake 35 %	2.4E + 01	2.68E + 01	11	2.15E + 01	−10	1.98E + 01	2.33E + 01
^131^I Iodine, Hippuran, normal renal function	5.2E-02	1.89E-02	−64	1.53E-02	−71	1.29E-02	1.78E-02
^131^I Iodine, labelled monoclonal antibodies, intact antibody	4.7E-01	4.40E-01	−6	3.59E-01	−24	3.26E-01	3.94E-01
^131^I Iodine NP59	1.8E + 00	2.02E + 00	12	1.74E + 00	−3	1.60E + 00	1.89E + 00
^201^Tl Thallium ion	1.4E-01	1.27E-01	−10	1.02E-01	−27	9.90E-02	1.05E-01

.

A new Supplemental file is given in a separate file named “Additional file 1: Table S1”.

## Additional file 1

Additional file 1: Table S1. Effective dose from all the radiopharmaceuticals published by the ICRP, determined using three different methods. (E/A_0_) 1 is the previously published effective dose per unit administered activity (E/A_0_) by ICRP, (E/A_0_)2 is (E/A_0_) dose calculated with the new phantoms and old tissue weighting factors while (E/A_0_) 3 is with the new phantoms and new weighting factors. (E/A_0_)2-(E/A_0_) 1))/ (E/A_0_)1 and ((E/A_0_) 3-(E/A_0_)1)/ (E/A_0_)1 is the difference in % of the new values compared to the old. (E/A_0_)3 male and (E/A_0_)3 are the effective dose estimations generated from the equivalent dose of each gender separately using the new phantoms and new weighting factors. (DOCX 65 kb)

