# Supplementary material for: Effective dose to adult patients from 338 radiopharmaceuticals estimated using ICRP biokinetic data, ICRP/ICRU computational reference phantoms and ICRP 2007 tissue weighting factors
Source: EJNMMI Phys. 2014 Sep 29;1:9. doi: 10.1186/2197-7364-1-9 (PMC4545621; doi:10.1186/2197-7364-1-9)
Supplement: Supplementary file 1 — Additional file 1: Table S1: Effective dose from all the radiopharmaceuticals published by the ICRP, determined using three different methods. (E/A0)1 is the previously published effective dose per unit administered activity (E/A0) by ICRP, (E/A0)2 is (E/A0) dose calculated with the new phantoms and old tissue weighting factors while (E/A0)3 is with the new phantoms and new weighting factors. (E/A0)2 − (E/A0)1))/(E/A0)1 and ((E/A0)3 − (E/A0)1)/(E/A0)1 is the difference in% of the new values compared to the old. (E/A0)3 male and (E/A0)3 are the estimations generated from the equivalent dose of each gender separately using the new phantoms and new weighting factors. (DOC 466 KB) [file 40658_2014_116_MOESM1_ESM.doc]

**Additional file 1: Table S1**. Effective dose from all the radiopharmaceuticals published by the ICRP, determined using three different methods. (E/A_0_) 1 is the previously published effective dose per unit administered activity (E/A_0_) by ICRP, (E/A_0_)2 is (E/A_0_) dose calculated with the new phantoms and old tissue weighting factors while (E/A_0_) 3 is with the new phantoms and new weighting factors. (E/A_0_)2-(E/A_0_) 1))/ (E/A_0_)1 and ((E/A_0_) 3-(E/A_0_)1)/ (E/A_0_)1 is the difference in % of the new values compared to the old. (E/A_0_)3 male and (E/A_0_)3 are the estimations generated from the equivalent dose of each gender separately using the new phantoms and new weighting factors.

| **Radiopharmaceuticals** | (E/A_0_)**1**  **[mSv/**  **MBq]** | (E/A_0_)**2**  **[mSv/**  **MBq]** | **(**(E/A_0_)**2-**(E/A_0_)**1) /**(E/A_0_)**1**  **[%]** | **(E/A_0_)3**  **[mSv/**  **MBq]** | **(**(E/A_0_)**3-**(E/A_0_)**1) /**(E/A_0_)**1**  **[%]** | (E/A_0_)**3 male**  **[mSv/**  **MBq]** | (E/A_0_)**3 female**  **[mSv/**  **MBq]** |
| --- | --- | --- | --- | --- | --- | --- | --- |
| **Phantom** | **MIRD** | **ICRP/**  **ICRU** |  | **ICRP/**  **ICRU** |  | **ICRP/**  **ICRU** | **ICRP/**  **ICRU** |
| **w_T_** | **ICRP**  **60** | **ICRP**  **60** |  | **ICRP 103** |  | **ICRP 103** | **ICRP 103** |
| As-72 Arsenate Arsenite | 3.6E-01 | 3.19E-01 | -11 | **3.45E-01** | -4 | 3.47E-01 | 3.43E-01 |
| As-74 Arsenate Arsenite | 5.1E-01 | 4.55E-01 | -11 | **5.06E-01** | -1 | 5.08E-01 | 5.03E-01 |
| As-76 Arsenate Arsenite | 2.8E-01 | 2.35E-01 | -16 | **2.47E-01** | -12 | 2.43E-01 | 2.51E-01 |
| Au-198 Gold colloid early to intermediate diffuse parenchymal liver disease | 1.4E+00 | 8.83E-01 | -37 | **4.93E-01** | -65 | 4.60E-01 | 5.27E-01 |
| Au-198 Gold colloid intermediate to advanced diffuse parenchymal liver disease | 1.7E+00 | 1.25E+00 | -26 | **6.36E-01** | -63 | 5.91E-01 | 6.81E-01 |
| Au-198 Gold colloid normal liver condition | 1.1E+00 | 5.59E-01 | -49 | **4.01E-01** | -64 | 3.80E-01 | 4.22E-01 |
| Ba-131 Barium | 5.0E-01 | 3.09E-01 | -38 | **3.34E-01** | -33 | 3.05E-01 | 3.64E-01 |
| Ba-131 Barium labelled non-absorbable markers orally administered of fluids | 4.9E-01 | 2.15E-01 | -56 | **2.22E-01** | -55 | 1.90E-01 | 2.54E-01 |
| Ba-131 Barium labelled non-absorbable markers orally administered of solids | 5.1E-01 | 2.23E-01 | -56 | **2.31E-01** | -55 | 1.97E-01 | 2.64E-01 |
| Ba-133m Barium | 4.7E-01 | 9.27E-02 | -80 | **1.03E-01** | -78 | 9.63E-02 | 1.09E-01 |
| Ba-135m Barium | 3.4E-01 | 6.96E-02 | -80 | **7.71E-02** | -77 | 7.33E-02 | 8.10E-02 |
| Br-76 Bromide | 2.8E-01 | 2.62E-01 | -6 | **2.96E-01** | 6 | 3.00E-01 | 2.92E-01 |
| Br-77 Bromide | 7.7E-02 | 6.89E-02 | -11 | **7.76E-02** | 1 | 7.93E-02 | 7.58E-02 |
| Br-77 Bromide Bromospiperone | 8.5E-02 | 8.03E-02 | -6 | **8.74E-02** | 3 | 8.47E-02 | 9.00E-02 |
| Br-82 Bromide | 4.0E-01 | 2.57E-02 | -94 | **2.92E-02** | -93 | 2.91E-02 | 2.93E-02 |
| C-11 Carbon (2-^11^C)thymidine | 2.7E-03 | 2.36E-03 | -13 | **2.53E-03** | -6 | 2.61E-03 | 2.45E-03 |
| C-11 Carbon (Methyl-^11^C)thymidine | 3.5E-03 | 3.10E-03 | -12 | **3.18E-03** | -9 | 3.18E-03 | 3.18E-03 |
| C-11 Carbon acetate | 3.5E-03 | 4.37E-03 | 25 | **4.20E-03** | 20 | 4.08E-03 | 4.31E-03 |
| C-11 Carbon amino acids | 5.6E-03 | 4.43E-03 | -21 | **4.62E-03** | -18 | 4.89E-03 | 4.34E-03 |
| C-11 Carbon brain receptor substances | 4.3E-03 | 3.22E-03 | -25 | **3.56E-03** | -17 | 3.69E-03 | 3.42E-03 |
| C-11 Carbon COHb-Labelled erythrocytes | 5.0E-03 | 3.38E-03 | -32 | **3.13E-03** | -37 | 4.78E-03 | 1.48E-03 |
| C-11 Carbon Dioxide continuous inhalation for 1 hr | 1.0E-03 | 9.27E-04 | -7 | **1.04E-03** | 4 | 1.04E-03 | 1.03E-03 |
| C-11 Carbon Dioxide single inhalation with 20 s breathhold | 1.6E-03 | 1.46E-03 | -9 | **1.59E-03** | -1 | 1.60E-03 | 1.58E-03 |
| C-11 Carbon Methionine | 8.4E-03 | 5.39E-03 | -36 | **5.49E-03** | -35 | 5.69E-03 | 5.28E-03 |
| C-11 Carbon Monoxide continuous inhalation for 1 hr | 3.2E-03 | 2.89E-03 | -10 | **2.46E-03** | -23 | 3.24E-03 | 1.68E-03 |
| C-11 Carbon Monoxide single inhale 20 s breathhold (2.5L) | 4.8E-03 | 4.39E-03 | -9 | **3.74E-03** | -22 | 4.94E-03 | 2.55E-03 |
| C-11 Carbon Realistic maximum | 1.1E-02 | 4.99E-03 | -55 | **5.46E-03** | -50 | 6.12E-03 | 4.79E-03 |
| C-11 Carbon Spiperone | 5.3E-03 | 4.55E-03 | -14 | **4.03E-03** | -24 | 3.88E-03 | 4.19E-03 |
| C-14 Carbon Inulin abnormal renal function | 1.5E-02 | 1.07E-02 | -29 | **1.17E-02** | -22 | 1.15E-02 | 1.18E-02 |
| C-14 Carbon Inulin normal renal function | 8.2E-03 | 1.33E-03 | -84 | **1.37E-03** | -83 | 1.34E-03 | 1.40E-03 |
| C-14 Carbon labelled neutral fat & free fatty acids | 2.1E+00 | 1.75E+00 | -17 | **2.76E+00** | 31 | 3.37E+00 | 2.16E+00 |
| C-14 Carbon labelled urea 14-C, helicobacter positive patient orally administered | 8.1E-02 | 7.50E-02 | -7 | **8.56E-02** | 6 | 8.54E-02 | 8.58E-02 |
| C-14 Carbon labelled urea 14-C, normal case orally administered | 3.1E-02 | 2.32E-02 | -25 | **2.65E-02** | -15 | 2.64E-02 | 2.66E-02 |
| Ca-45 Calcium, intravenous | 3.1E+00 | 1.34E-01 | -96 | **1.52E-01** | -95 | 1.51E-01 | 1.52E-01 |
| Ca-45 Calcium, orally | 1.8E+00 | 9.71E-02 | -95 | **1.11E-01** | -94 | 1.07E-01 | 1.15E-01 |
| Ca-47 Calcium, intravenous | 1.2E+00 | 4.15E-01 | -65 | **4.81E-01** | -60 | 4.80E-01 | 4.82E-01 |
| Ca-47 Calcium, orally | 1.8E+00 | 5.25E-01 | -71 | **5.78E-01** | -68 | 5.37E-01 | 6.19E-01 |
| Cl-34m Chloride (47% of Cl-34 decays) | 1.4E-02 | 9.74E-03 | -30 | **1.10E-02** | -21 | 1.12E-02 | 1.09E-02 |
| Cl-36 Chloride | 6.7E-01 | 7.20E-01 | 7 | **8.20E-01** | 22 | 8.15E-01 | 8.25E-01 |
| Cl-38 Chloride | 1.4E-02 | 1.33E-02 | -5 | **1.51E-02** | 8 | 1.51E-02 | 1.50E-02 |
| Co-57 Cobalt labelled bleomycin | 4.7E-02 | 1.41E-02 | -70 | **1.61E-02** | -66 | 1.64E-02 | 1.57E-02 |
| Co-57 Cobalt Vitamin B12, intravenous with carrier | 4.6E-01 | 3.42E-01 | -26 | **4.12E-01** | -10 | 4.16E-01 | 4.09E-01 |
| Co-57 Cobalt Vitamin B12, intravenous without carrier | 4.4E+00 | 3.07E+00 | -30 | **3.80E+00** | -14 | 3.81E+00 | 3.80E+00 |
| Co-57 Cobalt Vitamin B12, orally with flushing | 2.1E+00 | 1.45E+00 | -31 | **1.78E+00** | -15 | 1.78E+00 | 1.77E+00 |
| Co-57 Cobalt Vitamin B12, orally without flushing | 3.1E+00 | 2.16E+00 | -30 | **2.66E+00** | -14 | 2.67E+00 | 2.66E+00 |
| Co-58 Cobalt Vitamin B12, intravenous without carrier | 8.2E+00 | 7.10E+00 | -13 | **8.89E+00** | 8 | 8.79E+00 | 8.99E+00 |
| Co-58 Cobalt Vitamin B12, intravenous with carrier | 8.9E-01 | 7.69E-01 | -14 | **9.41E-01** | 6 | 9.42E-01 | 9.39E-01 |
| Co-58 Cobalt Vitamin B12, orally with flushing | 4.0E+00 | 3.39E+00 | -15 | **4.21E+00** | 5 | 4.17E+00 | 4.26E+00 |
| Co-58 Cobalt Vitamin B12, orally without flushing | 5.9E+00 | 5.04E+00 | -15 | **6.28E+00** | 6 | 6.21E+00 | 6.35E+00 |
| Cr-51 Chromium(III) Chloride | 6.8E-02 | 6.72E-02 | -1 | **7.13E-02** | 5 | 7.05E-02 | 7.21E-02 |
| Cr-51 Chromium EDTA, intravenous abnormal renal function | 4.6E-03 | 4.11E-03 | -11 | **4.57E-03** | -1 | 4.78E-03 | 4.36E-03 |
| Cr-51 Chromium EDTA, intravenous normal renal function | 2.0E-03 | 1.39E-03 | -31 | **1.56E-03** | -22 | 1.76E-03 | 1.36E-03 |
| Cr-51 Chromium EDTA, orally | 4.4E-02 | 1.42E-02 | -68 | **1.46E-02** | -67 | 1.25E-02 | 1.67E-02 |
| Cr-51 Chromium labelled denatured erythrocytes | 1.8E-01 | 2.47E-01 | 37 | **1.35E-01** | -25 | 1.25E-01 | 1.45E-01 |
| Cr-51 Chromium labelled erythrocytes | 1.7E-01 | 1.21E-01 | -29 | **9.92E-02** | -42 | 9.56E-02 | 1.03E-01 |
| Cr-51 Chromium labelled non-absorbable markers, orally administered of fluids | 4.3E-02 | 1.42E-02 | -67 | **1.46E-02** | -66 | 1.25E-02 | 1.67E-02 |
| Cr-51 Chromium labelled non-absorbable markers, orally administered of solids | 4.5E-02 | 1.47E-02 | -67 | **1.52E-02** | -66 | 1.30E-02 | 1.74E-02 |
| Cr-51 Chromium labelled platelets | 1.4E-01 | 1.39E-01 | -1 | **9.33E-02** | -33 | 8.74E-02 | 9.91E-02 |
| Cr-51 Chromium labelled white blood cells (leukocytes) | 1.2E-01 | 1.06E-01 | -12 | **7.44E-02** | -38 | 6.97E-02 | 7.91E-02 |
| Cs-129 Caesium | 4.9E-02 | 3.59E-02 | -27 | **4.00E-02** | -18 | 4.11E-02 | 3.88E-02 |
| Cs-130 Caesium | 3.4E-03 | 4.20E-04 | -88 | **4.78E-04** | -86 | 4.99E-04 | 4.58E-04 |
| Cs-131 Caesium | 5.0E-02 | 3.54E-02 | -29 | **3.86E-02** | -23 | 3.93E-02 | 3.79E-02 |
| Cs-134 Caesium | 2.5E-03 | 2.09E-03 | -16 | **2.35E-03** | -6 | 2.43E-03 | 2.28E-03 |
| Cs-134m Caesium | 6.7E-03 | 2.04E-03 | -70 | **2.37E-03** | -65 | 2.49E-03 | 2.25E-03 |
| Cu-64 Copper | 3.6E-02 | 2.11E-02 | -41 | **2.36E-02** | -34 | 2.31E-02 | 2.41E-02 |
| Cu-67 Copper | 1.5E-01 | 8.49E-02 | -43 | **9.03E-02** | -40 | 8.93E-02 | 9.14E-02 |
| F-18 Fluoride | 2.4E-02 | 1.19E-02 | -50 | **1.34E-02** | -44 | 1.44E-02 | 1.23E-02 |
| F-18 Fluoride L-dopa | 2.5E-02 | 1.51E-02 | -40 | **1.68E-02** | -33 | 1.85E-02 | 1.52E-02 |
| F-18 Fluoride FDG | 1.9E-02 | 1.50E-02 | -21 | **1.59E-02** | -16 | 1.66E-02 | 1.53E-02 |
| F-18 Fluoride labelled amino acids | 2.3E-02 | 1.75E-02 | -24 | **1.86E-02** | -19 | 1.97E-02 | 1.74E-02 |
| F-18 Fluoride labelled brain receptor sub | 2.8E-02 | 1.89E-02 | -33 | **1.91E-02** | -32 | 1.93E-02 | 1.89E-02 |
| Fe-52 Iron, intravenous | 1.1E+00 | 3.22E-02 | -97 | **3.93E-02** | -96 | 3.70E-02 | 4.17E-02 |
| Fe-52 Iron, orally | 7.1E-01 | 7.78E-02 | -89 | **8.33E-02** | -88 | 8.02E-02 | 8.64E-02 |
| Fe-55 Iron, intravenous | 4.0E+00 | 1.58E+00 | -61 | **1.09E+00** | -73 | 1.06E+00 | 1.11E+00 |
| Fe-55 Iron, orally | 4.2E-01 | 1.63E-01 | -61 | **1.13E-01** | -73 | 1.10E-01 | 1.15E-01 |
| Fe-59 Iron, intravenous | 1.0E+01 | 4.65E+00 | -54 | **4.91E+00** | -51 | 4.73E+00 | 5.09E+00 |
| Fe-59 Iron, orally | 2.0E+00 | 8.61E-01 | -57 | **9.03E-01** | -55 | 8.26E-01 | 9.81E-01 |
| Ga-66 Gallium citrate | 3.2E-01 | 2.11E-01 | -34 | **2.38E-01** | -26 | 2.37E-01 | 2.39E-01 |
| Ga-67 Gallium citrate | 1.0E-01 | 7.66E-02 | -23 | **8.59E-02** | -14 | 8.58E-02 | 8.59E-02 |
| Ga-68 Gallium citrate | 2.0E-02 | 1.60E-02 | -20 | **1.80E-02** | -10 | 1.79E-02 | 1.80E-02 |
| Ga-68 Gallium labelled EDTA | 4.0E-02 | 2.35E-02 | -41 | **2.37E-02** | -41 | 2.45E-02 | 2.29E-02 |
| Ga-72 Gallium citrate | 3.4E-01 | 2.47E-01 | -27 | **2.77E-01** | -19 | 2.76E-01 | 2.78E-01 |
| H-3 Tritium Inulin abnormal renal function | 1.7E-03 | 1.24E-03 | -27 | **1.34E-03** | -21 | 1.32E-03 | 1.36E-03 |
| H-3 Tritium Inulin normal renal function | 9.4E-04 | 1.50E-04 | -84 | **1.54E-04** | -84 | 1.51E-04 | 1.58E-04 |
| H-3 Tritium labelled neutral fat & free fatty acids | 2.2E-01 | 9.34E-02 | -58 | **1.72E-01** | -22 | 2.38E-01 | 1.05E-01 |
| H-3 Tritium water | 1.5E-02 | 1.30E-02 | -13 | **1.48E-02** | -1 | 1.64E-02 | 1.32E-02 |
| Hg-197 Mercury BMHP | 1.4E-01 | 3.11E-01 | 122 | **2.69E-01** | 92 | 2.53E-01 | 2.84E-01 |
| Hg-197 Mercury Chlormerodrin | 8.7E-02 | 1.34E-01 | 54 | **1.14E-01** | 31 | 1.08E-01 | 1.20E-01 |
| Hg-197 Mercury Dichloride | 1.4E-01 | 2.98E-01 | 113 | **2.54E-01** | 81 | 2.40E-01 | 2.68E-01 |
| Hg-203 Mercury Chlormerodrin | 1.1E+00 | 1.29E+00 | 17 | **1.16E+00** | 5 | 1.11E+00 | 1.22E+00 |
| I-123 Iodine BMIPP | 1.6E-02 | 1.37E-02 | -14 | **1.57E-02** | -2 | 1.62E-02 | 1.52E-02 |
| I-123 Iodine HIPPURAN abnormal renal function | 9.8E-03 | 8.46E-03 | -14 | **9.17E-03** | -6 | 1.01E-02 | 8.26E-03 |
| I-123 Iodine HIPPURAN normal renal function | 1.2E-02 | 7.41E-03 | -38 | **8.32E-03** | -31 | 1.00E-02 | 6.62E-03 |
| I-123 Iodine HIPPURAN Unilateral renal blockage abnormal kidney | 3.0E-02 | 5.70E-02 | 90 | **5.02E-02** | 67 | 4.78E-02 | 5.25E-02 |
| I-123 Iodine HIPPURAN Unilateral renal blockage normal kidney | 1.3E-02 | 1.05E-02 | -19 | **1.19E-02** | -8 | 1.30E-02 | 1.08E-02 |
| I-123 Iodine IMP Bound iodine | 2.7E-02 | 2.56E-02 | -5 | **2.11E-02** | -22 | 1.96E-02 | 2.25E-02 |
| I-123 Iodine IMP Released iodine | 2.8E-02 | 2.65E-02 | -5 | **2.21E-02** | -21 | 2.08E-02 | 2.33E-02 |
| I-123 Iodine IPPA | 1.6E-02 | 1.38E-02 | -14 | **1.58E-02** | -1 | 1.63E-02 | 1.53E-02 |
| I-123 Iodine labelled Albumin HSA | 2.0E-02 | 4.75E-03 | -76 | **5.08E-03** | -75 | 4.95E-03 | 5.21E-03 |
| I-123 Iodine labelled albumin (intrathecal administered) cisternal injection | 1.8E-02 | 1.39E-03 | -92 | **1.49E-03** | -92 | 1.45E-03 | 1.52E-03 |
| I-123 Iodine labelled albumin (intrathecal administered) lumbar injection | 1.8E-02 | 1.79E-03 | -90 | **1.92E-03** | -89 | 1.87E-03 | 1.97E-03 |
| I-123 Iodine labelled brain receptor sub | 5.0E-02 | 3.33E-02 | -33 | **3.30E-02** | -34 | 3.18E-02 | 3.43E-02 |
| I-123 Iodine labelled fibrinogen bound iodine | 2.0E-02 | 5.44E-03 | -73 | **5.82E-03** | -71 | 5.67E-03 | 5.96E-03 |
| I-123 Iodine labelled fibrinogen released iodine | 1.3E-03 | 1.04E-03 | -20 | **1.16E-03** | -11 | 1.26E-03 | 1.06E-03 |
| I-123 Iodine labelled MAA, early to intermediate diffuse parenchymal liver disease | 1.9E-02 | 1.78E-02 | -6 | **1.57E-02** | -17 | 1.58E-02 | 1.55E-02 |
| I-123 Iodine labelled MAA, intermediate to advanced diffuse parenchymal liver disease | 2.2E-02 | 2.18E-02 | -1 | **1.72E-02** | -22 | 1.71E-02 | 1.73E-02 |
| I-123 Iodine labelled MAA, normal condition | 1.8E-02 | 1.43E-02 | -21 | **1.44E-02** | -20 | 1.47E-02 | 1.41E-02 |
| I-123 Iodine labelled monoclonal antibodies Fab fragments | 2.4E-02 | 2.34E-02 | -3 | **2.27E-02** | -5 | 2.24E-02 | 2.30E-02 |
| I-123 Iodine labelled monoclonal antibodies Fab2 fragments | 2.5E-02 | 2.18E-02 | -13 | **2.26E-02** | -10 | 2.22E-02 | 2.31E-02 |
| I-123 Iodine labelled monoclonal antibodies intact antibody | 2.9E-02 | 2.33E-02 | -20 | **2.18E-02** | -25 | 1.11E-03 | 1.24E-03 |
| I-123 Iodine MIBG | 1.3E-02 | 1.14E-02 | -12 | **1.32E-02** | 2 | 1.36E-02 | 1.27E-02 |
| I-123 Iodine Sodium rose Bengal, normal hepato-biliary condition | 5.9E-02 | 2.92E-02 | -51 | **3.17E-02** | -46 | 2.99E-02 | 3.34E-02 |
| I-123 Iodine Sodium rose Bengal, occlusion of the common bile duct | 2.5E-02 | 1.45E-02 | -42 | **1.88E-02** | -25 | 1.86E-02 | 1.90E-02 |
| I-123 Iodine Sodium rose Bengal, occlusion of the cystic duct | 4.5E-02 | 2.37E-02 | -47 | **2.58E-02** | -43 | 2.50E-02 | 2.65E-02 |
| I-123 Iodine Sodium rose Bengal, parenchymal liver disease | 2.7E-02 | 1.46E-02 | -46 | **1.63E-02** | -40 | 1.68E-02 | 1.57E-02 |
| I-123 Iodide Thyroid block uptake 0% | 1.1E-02 | 8.69E-03 | -21 | **9.70E-03** | -12 | 1.05E-02 | 8.86E-03 |
| I-123 Iodide Thyroid uptake 5% | 4.7E-02 | 4.96E-02 | 6 | **4.48E-02** | -5 | 4.26E-02 | 4.71E-02 |
| I-123 Iodide Thyroid uptake 15% | 1.1E-01 | 1.25E-01 | 14 | **1.08E-01** | -2 | 9.95E-02 | 1.16E-01 |
| I-123 Iodide Thyroid uptake 25% | 1.7E-01 | 1.98E-01 | 16 | **1.70E-01** | 0 | 1.56E-01 | 1.84E-01 |
| I-123 Iodide Thyroid uptake 35% | 2.2E-01 | 2.72E-01 | 24 | **2.33E-01** | 6 | 2.12E-01 | 2.53E-01 |
| I-123 Iodide Thyroid uptake 45% | 2.8E-01 | 3.46E-01 | 24 | **2.95E-01** | 5 | 2.69E-01 | 3.22E-01 |
| I-123 Iodide Thyroid uptake 55% | 3.4E-01 | 4.21E-01 | 24 | **3.58E-01** | 5 | 3.26E-01 | 3.91E-01 |
| I-124 Iodide Thyroid block uptake 0% | 9.5E-02 | 7.59E-02 | -20 | **8.38E-02** | -12 | 8.93E-02 | 7.83E-02 |
| I-124 Iodide Thyroid uptake 5% | 2.2E+00 | 2.23E+00 | 1 | **1.91E+00** | -13 | 1.73E+00 | 2.10E+00 |
| I-124 Iodide Thyroid uptake 15% | 6.4E+00 | 6.50E+00 | 2 | **5.52E+00** | -14 | 4.95E+00 | 6.09E+00 |
| I-124 Iodide Thyroid uptake 25% | 1.1E+01 | 1.08E+01 | -2 | **9.15E+00** | -17 | 8.20E+00 | 1.01E+01 |
| I-124 Iodide Thyroid uptake 35% | 1.5E+01 | 1.51E+01 | 1 | **1.28E+01** | -15 | 1.14E+01 | 1.41E+01 |
| I-124 Iodide Thyroid uptake 45% | 1.9E+01 | 1.93E+01 | 2 | **1.64E+01** | -14 | 1.46E+01 | 1.81E+01 |
| I-124 Iodide Thyroid uptake 55% | 2.3E+01 | 2.36E+01 | 3 | **2.00E+01** | -13 | 1.79E+01 | 2.21E+01 |
| I-125 Iodine Diiodothyronine | 3.6E-02 | 2.07E-02 | -43 | **2.20E-02** | -39 | 2.02E-02 | 2.38E-02 |
| I-125 Iodine HIPPURAN abnormal renal function | 7.0E-03 | 6.77E-03 | -3 | **7.07E-03** | 1 | 7.71E-03 | 6.44E-03 |
| I-125 Iodine HIPPURAN normal renal function | 7.7E-03 | 5.13E-03 | -33 | **5.54E-03** | -28 | 6.59E-03 | 4.49E-03 |
| I-125 Iodine HIPPURAN Unilateral renal blockage Abnormal kidney | 1.3E-01 | 3.43E-01 | 164 | **2.88E-01** | 122 | 2.69E-01 | 3.08E-01 |
| I-125 Iodine HIPPURAN Unilateral renal blockage normal kidney | 3.6E-02 | 3.56E-02 | -1 | **3.96E-02** | 10 | 4.08E-02 | 3.83E-02 |
| I-125 Iodine iodiantipyrine | 1.0E-02 | 9.12E-03 | -9 | **9.93E-03** | -1 | 1.06E-02 | 9.22E-03 |
| I-125 Iodine iodinated PVP | 6.5E-01 | 4.99E-01 | -23 | **5.68E-01** | -13 | 5.36E-01 | 6.00E-01 |
| I-125 Iodine iothalamate abnormal renal function | 1.4E-02 | 1.47E-02 | 5 | **1.58E-02** | 13 | 1.65E-02 | 1.51E-02 |
| I-125 Iodine iothalamate normal renal function | 7.2E-03 | 5.62E-03 | -22 | **6.07E-03** | -16 | 6.85E-03 | 5.28E-03 |
| I-125 iodine labelled Albumin HSA | 2.2E-01 | 4.79E-02 | -78 | **4.95E-02** | -78 | 4.76E-02 | 5.15E-02 |
| I-125 iodine labelled fibrinogen bound iodine | 8.0E-02 | 2.29E-02 | -71 | **2.37E-02** | -70 | 2.27E-02 | 2.46E-02 |
| I-125 iodine labelled fibrinogen released iodine | 8.0E-03 | 7.26E-03 | -9 | **7.90E-03** | -1 | 8.51E-03 | 7.29E-03 |
| I-125 Iodine labelled non-absorbable markers, orally administered of fluids | 1.7E-01 | 6.22E-02 | -63 | **6.41E-02** | -62 | 5.24E-02 | 7.58E-02 |
| I-125 Iodine labelled non-absorbable markers, orally administered of solids | 1.7E-01 | 6.45E-02 | -62 | **6.65E-02** | -61 | 5.42E-02 | 7.88E-02 |
| I-125 Iodine Reverse Triiodothyronine (rT3) | 3.7E-02 | 2.13E-02 | -42 | **2.27E-02** | -39 | 2.09E-02 | 2.45E-02 |
| I-125 Iodide Thyroid block uptake 0% | 9.1E-03 | 7.98E-03 | -12 | **8.68E-03** | -5 | 9.38E-03 | 7.98E-03 |
| I-125 Iodide Thyroid uptake 5% | 2.1E+00 | 2.83E+00 | 35 | **2.39E+00** | 14 | 2.16E+00 | 2.62E+00 |
| I-125 Iodide Thyroid uptake 15% | 6.2E+00 | 8.48E+00 | 37 | **7.15E+00** | 15 | 6.45E+00 | 7.85E+00 |
| I-125 Iodide Thyroid uptake 25% | 1.0E+01 | 1.41E+01 | 41 | **1.19E+01** | 19 | 1.07E+01 | 1.31E+01 |
| I-125 Iodide Thyroid uptake 35% | 1.4E+01 | 1.98E+01 | 41 | **1.66E+01** | 19 | 1.50E+01 | 1.83E+01 |
| I-125 Iodide Thyroid uptake 45% | 1.9E+01 | 2.54E+01 | 34 | **2.15E+01** | 13 | 1.94E+01 | 2.36E+01 |
| I-125 Iodide Thyroid uptake 55% | 2.3E+01 | 3.11E+01 | 35 | **2.62E+01** | 14 | 2.36E+01 | 2.87E+01 |
| I-125 Iodine Thyroxine | 1.0E-01 | 9.34E-02 | -7 | **1.03E-01** | 3 | 1.02E-01 | 1.04E-01 |
| I-125 Iodine Triiodothyronine (T3) | 4.7E-02 | 3.32E-02 | -29 | **3.60E-02** | -23 | 3.44E-02 | 3.76E-02 |
| I-131 Iodine Diiodothyronine | 2.5E-01 | 7.77E-02 | -69 | **8.54E-02** | -66 | 8.16E-02 | 8.92E-02 |
| I-131 Iodine HIPPURAN, abnormal renal function | 4.8E-02 | 3.07E-02 | -36 | **3.24E-02** | -33 | 3.41E-02 | 3.06E-02 |
| I-131 Iodine HIPPURAN, normal renal function | 5.2E-02 | 1.65E-02 | -68 | **1.80E-02** | -65 | 2.10E-02 | 1.51E-02 |
| I-131 Iodine HIPPURAN, unilateral renal blockage abnormal kidney | 6.6E-01 | 1.29E+00 | 95 | **1.07E+00** | 62 | 1.00E+00 | 1.14E+00 |
| I-131 Iodine HIPPURAN, unilateral renal blockage normal kidney | 1.8E-01 | 1.43E-01 | -21 | **1.63E-01** | -9 | 1.68E-01 | 1.59E-01 |
| I-131 Iodine Iodiantipyrine | 6.7E-02 | 4.48E-02 | -33 | **5.02E-02** | -25 | 5.25E-02 | 4.80E-02 |
| I-131 Iodine iodinated PVP | 6.0E-01 | 4.30E-01 | -28 | **4.78E-01** | -20 | 4.63E-01 | 4.93E-01 |
| I-131 Iodine labelled Albumin HSA | 6.4E-01 | 1.19E-01 | -81 | **1.25E-01** | -80 | 1.24E-01 | 1.26E-01 |
| I-131 Iodine labelled albumin (intrathecal administered) cisternal injection | 7.2E-01 | 1.02E-01 | -86 | **1.07E-01** | -85 | 1.06E-01 | 1.07E-01 |
| I-131 Iodine labelled albumin (intrathecal administered) lumbar injection | 3.0E-01 | 1.07E-01 | -65 | **1.12E-01** | -63 | 1.11E-01 | 1.13E-01 |
| I-131 Iodine labelled fibrinogen bound iodine | 4.2E-01 | 9.75E-02 | -77 | **1.02E-01** | -76 | 1.02E-01 | 1.03E-01 |
| I-131 Iodine labelled fibrinogen released iodine | 4.0E-02 | 2.55E-02 | -36 | **2.86E-02** | -29 | 3.00E-02 | 2.71E-02 |
| I-131 Iodine labelled MAA | 4.5E-01 | 3.66E-01 | -19 | **2.54E-01** | -44 | 2.36E-01 | 2.71E-01 |
| I-131 Iodine labelled microaggregated albumin early to intermediate diffuse parenchymal liver disease | 2.4E-01 | 1.98E-01 | -18 | **1.41E-01** | -41 | 1.38E-01 | 1.45E-01 |
| I-131 Iodine labelled microaggregated albumin intermediate to advanced diffuse parenchymal liver disease | 2.9E-01 | 2.59E-01 | -11 | **1.64E-01** | -43 | 1.58E-01 | 1.69E-01 |
| I-131 Iodine labelled microaggregated albumin normal condition | 2.2E-01 | 1.37E-01 | -38 | **1.18E-01** | -46 | 1.17E-01 | 1.19E-01 |
| I-131 Iodine labelled monoclonal antibodies, Fab fragments | 1.7E-01 | 1.31E-01 | -23 | **1.24E-01** | -27 | 1.22E-01 | 1.26E-01 |
| I-131 Iodine labelled monoclonal antibodies, Fab2 fragments | 2.0E-01 | 1.45E-01 | -28 | **1.46E-01** | -27 | 1.43E-01 | 1.50E-01 |
| I-131 Iodine labelled monoclonal antibodies, intact antibody | 4.7E-01 | 3.13E-01 | -33 | **2.57E-01** | -45 | 2.49E-01 | 2.66E-01 |
| I-131 Iodine labelled non-absorbable markers, orally administered of fluids | 1.2E+00 | 1.53E-01 | -87 | **1.59E-01** | -87 | 1.37E-01 | 1.80E-01 |
| I-131 Iodine labelled non-absorbable markers, orally administered of solids | 1.2E+00 | 1.59E-01 | -87 | **1.64E-01** | -86 | 1.42E-01 | 1.87E-01 |
| I-131 Iodine MIGB | 1.4E-01 | 1.04E-01 | -26 | **1.05E-01** | -25 | 1.05E-01 | 1.05E-01 |
| I-131 Iodine NP-59 | 1.8E+00 | 1.94E+00 | 8 | **1.73E+00** | -4 | 1.62E+00 | 1.84E+00 |
| I-131 Iodine Reverse Triiodothyronine (rT3) | 2.5E-01 | 8.10E-02 | -68 | **8.92E-02** | -64 | 8.55E-02 | 9.29E-02 |
| I-131 Iodine Sodium rose Bengal, normal hepato-biliary condition | 1.1E+00 | 1.48E-01 | -87 | **1.56E-01** | -86 | 1.59E-01 | 1.53E-01 |
| I-131 Iodine Sodium rose Bengal, occlusion of the common bile duct | 6.4E-01 | 2.29E-01 | -64 | **2.96E-01** | -54 | 2.89E-01 | 3.03E-01 |
| I-131 Iodine Sodium rose Bengal, occlusion of the cystic duct | 5.5E-01 | 1.12E-01 | -80 | **1.17E-01** | -79 | 1.03E-01 | 1.31E-01 |
| I-131 Iodine Sodium rose Bengal, parenchymal liver disease | 3.0E-01 | 6.63E-02 | -78 | **7.04E-02** | -77 | 6.49E-02 | 7.60E-02 |
| I-131 Iodide Thyroid block uptake 0% | 6.1E-02 | 3.82E-02 | -37 | **4.27E-02** | -30 | 4.49E-02 | 4.06E-02 |
| I-131 Iodide Thyroid uptake 5% | 3.6E+00 | 3.91E+00 | 9 | **3.20E+00** | -11 | 2.93E+00 | 3.47E+00 |
| I-131 Iodide Thyroid uptake 15% | 1.1E+01 | 1.17E+01 | 6 | **9.56E+00** | -13 | 8.74E+00 | 1.04E+01 |
| I-131 Iodide Thyroid uptake 25% | 1.7E+01 | 1.95E+01 | 15 | **1.59E+01** | -6 | 1.45E+01 | 1.72E+01 |
| I-131 Iodide Thyroid uptake 35% | 2.4E+01 | 2.72E+01 | 13 | **2.22E+01** | -8 | 2.03E+01 | 2.41E+01 |
| I-131 Iodide Thyroid uptake 45% | 3.1E+01 | 3.51E+01 | 13 | **2.86E+01** | -8 | 2.61E+01 | 3.10E+01 |
| I-131 Iodide Thyroid uptake 55% | 3.8E+01 | 4.28E+01 | 13 | **3.49E+01** | -8 | 3.19E+01 | 3.79E+01 |
| I-131 Iodine Thyroxine | 4.4E-01 | 3.34E-01 | -24 | **3.82E-01** | -13 | 3.86E-01 | 3.79E-01 |
| I-131 Iodine Triiodothyronine (T3) | 3.0E-01 | 1.42E-01 | -53 | **1.59E-01** | -47 | 1.57E-01 | 1.61E-01 |
| In-111 Indium | 2.1E-01 | 1.71E-01 | -19 | **1.91E-01** | -9 | 1.85E-01 | 1.97E-01 |
| In-111 Indium DTPA, abnormal renal function | 4.2E-02 | 3.88E-02 | -8 | **4.31E-02** | 3 | 4.57E-02 | 4.05E-02 |
| In-111 Indium DTPA, (intrathecal administered) cisternal injection | 6.3E-02 | 5.50E-02 | -13 | **6.22E-02** | -1 | 6.58E-02 | 5.87E-02 |
| In-111 Indium DTPA, (intrathecal administered) lumbar injection | 5.5E-02 | 4.69E-02 | -15 | **5.30E-02** | -4 | 5.63E-02 | 4.97E-02 |
| In-111 Indium DTPA, normal renal function | 2.1E-02 | 1.54E-02 | -27 | **1.73E-02** | -18 | 2.01E-02 | 1.46E-02 |
| In-111 Indium labelled aerosols Substance with fast clearance from lungs | 2.5E-02 | 1.61E-02 | -36 | **1.87E-02** | -25 | 2.10E-02 | 1.65E-02 |
| In-111 Indium labelled aerosols Substance with slow clearance from lungs | 2.4E-01 | 3.26E-01 | 36 | **2.75E-01** | 15 | 2.52E-01 | 2.99E-01 |
| In-111 Indium labelled bleomycin | 1.0E-01 | 1.22E-01 | 22 | **1.27E-01** | 27 | 1.25E-01 | 1.29E-01 |
| In-111 Indium labelled HIG | 1.7E-01 | 1.39E-01 | -18 | **1.41E-01** | -17 | 1.44E-01 | 1.38E-01 |
| In-111 Indium labelled monoclonal antibodies, Fab fragments | 3.7E-01 | 1.96E-01 | -47 | **2.13E-01** | -42 | 2.06E-01 | 2.21E-01 |
| In-111 Indium labelled monoclonal antibodies, Fab2 fragments | 3.6E-01 | 1.95E-01 | -46 | **2.15E-01** | -40 | 2.08E-01 | 2.23E-01 |
| In-111 Indium labelled monoclonal antibodies, intact antibody | 3.3E-01 | 2.14E-01 | -35 | **2.24E-01** | -32 | 2.17E-01 | 2.32E-01 |
| In-111 Indium labelled non-absorbable markers, orally administered of fluids | 3.1E-01 | 1.49E-01 | -52 | **1.56E-01** | -50 | 1.37E-01 | 1.74E-01 |
| In-111 Indium labelled non-absorbable markers, orally administered of solids | 3.2E-01 | 1.55E-01 | -52 | **1.62E-01** | -49 | 1.42E-01 | 1.81E-01 |
| In-111 Indium labelled plateletes (thrombocytes) | 3.9E-01 | 4.31E-01 | 11 | **2.95E-01** | -24 | 2.76E-01 | 3.15E-01 |
| In-111 Indium labelled white blood cells (leukocytes) | 3.6E-01 | 3.71E-01 | 3 | **2.82E-01** | -22 | 2.67E-01 | 2.98E-01 |
| In-111 Indium octreotide | 5.4E-02 | 8.02E-02 | 49 | **6.87E-02** | 27 | 6.79E-02 | 6.96E-02 |
| In-113m Indium | 1.0E-02 | 6.00E-03 | -40 | **6.24E-03** | -38 | 6.01E-03 | 6.48E-03 |
| In-113m Indium (colloidal) early to intermediate parenchymal liver disease | 1.4E-02 | 1.12E-02 | -20 | **6.94E-03** | -50 | 6.52E-03 | 7.37E-03 |
| In-113m Indium (colloidal) intermediate to advanced diffuse parenchymal liver disease | 1.8E-02 | 1.53E-02 | -15 | **8.47E-03** | -53 | 7.88E-03 | 9.06E-03 |
| In-113m Indium (colloidal) normal liver condition | 1.1E-02 | 6.93E-03 | -37 | **5.33E-03** | -52 | 5.05E-03 | 5.61E-03 |
| In-113m Indium DTPA, abnormal renal function | 5.5E-03 | 4.87E-03 | -11 | **5.35E-03** | -3 | 5.47E-03 | 5.23E-03 |
| In-113m Indium DTPA, normal renal function | 1.1E-02 | 5.78E-03 | -47 | **6.15E-03** | -44 | 6.66E-03 | 5.64E-03 |
| In-113m Indium labelled aerosols Substance with fast clearance from lungs | 1.6E-02 | 4.42E-03 | -72 | **5.06E-03** | -68 | 5.29E-03 | 4.83E-03 |
| In-113m Indium labelled aerosols Substance with slow clearance from lungs | 2.5E-02 | 2.58E-02 | 3 | **1.45E-02** | -42 | 1.28E-02 | 1.61E-02 |
| In-113m Indium labelled non-absorbable markers, orally administered of fluids | 2.0E-02 | 6.74E-03 | -66 | **7.17E-03** | -64 | 6.99E-03 | 7.35E-03 |
| In-113m Indium labelled non-absorbable markers, orally administered of solids | 2.9E-02 | 7.74E-03 | -73 | **8.19E-03** | -72 | 7.86E-03 | 8.52E-03 |
| K-38 Potassium ultrashort lived | 1.9E-02 | 1.98E-02 | 4 | **1.86E-02** | -2 | 1.72E-02 | 2.01E-02 |
| K-42 Potassium, intravenous | 2.8E-01 | 1.00E-01 | -64 | **1.20E-01** | -57 | 1.21E-01 | 1.20E-01 |
| K-42 Potassium, orally | 3.4E-01 | 1.74E-01 | -49 | **2.05E-01** | -40 | 2.07E-01 | 2.03E-01 |
| K-43 Potassium, intravenous | 2.0E-01 | 1.10E-01 | -45 | **1.26E-01** | -37 | 1.29E-01 | 1.22E-01 |
| K-43 Potassium, orally | 2.2E-01 | 1.16E-01 | -47 | **1.33E-01** | -40 | 1.36E-01 | 1.29E-01 |
| Kr-81m Krypton | 2.7E-05 | 2.74E-05 | 1 | **1.52E-05** | -44 | 1.36E-05 | 1.68E-05 |
| La-140 Lanthanum DTPA, abnormal renal function | 2.6E-01 | 2.21E-01 | -15 | **2.43E-01** | -7 | 2.52E-01 | 2.35E-01 |
| La-140Lanthanum DTPA, normal renal function | 1.5E-01 | 8.98E-02 | -40 | **9.68E-02** | -35 | 1.07E-01 | 8.63E-02 |
| Mg-28 Magnesium | 7.2E-01 | 9.86E-02 | -86 | **1.15E-01** | -84 | 1.15E-01 | 1.15E-01 |
| N-13 Nitrogen Ammonia | 2.0E-03 | 8.04E-04 | -60 | **7.14E-04** | -64 | 8.13E-04 | 6.16E-04 |
| N-13 Nitrogen gas continuous inhalation for 1 hr | 4.3E-04 | 4.34E-04 | 1 | **2.45E-04** | -43 | 2.16E-04 | 2.74E-04 |
| N-13 Nitrogen gas single inhalation with 20 s breathhold | 3.8E-04 | 3.87E-04 | 2 | **2.19E-04** | -42 | 1.93E-04 | 2.44E-04 |
| N-13 Nitrogen gas solution | 4.1E-04 | 4.10E-04 | 0 | **2.42E-04** | -41 | 2.16E-04 | 2.68E-04 |
| N-13 Nitrogen L-glutamate | 3.9E-03 | 1.72E-03 | -56 | **2.85E-03** | -27 | 2.70E-03 | 3.01E-03 |
| Na-22 Sodium, intravenous | 2.0E+00 | 2.42E+00 | 21 | **2.78E+00** | 39 | 2.83E+00 | 2.74E+00 |
| Na-22 Sodium, orally | 2.6E+00 | 2.43E+00 | -7 | **2.77E+00** | 7 | 2.82E+00 | 2.72E+00 |
| Na-24 Sodium, intravenous | 3.2E-01 | 2.41E-01 | -25 | **2.76E-01** | -14 | 2.80E-01 | 2.72E-01 |
| Na-24 Sodium, orally | 3.6E-01 | 2.59E-01 | -28 | **2.93E-01** | -19 | 2.97E-01 | 2.89E-01 |
| O-15 Oxygen Carbon Dioxide, continuous inhalation 1 hr | 3.8E-04 | 3.51E-04 | -8 | **3.60E-04** | -5 | 3.56E-04 | 3.63E-04 |
| O-15 Oxygen Carbon Dioxide, single inhalation with 20 s breathold | 5.1E-04 | 4.75E-04 | -7 | **4.86E-04** | -5 | 4.81E-04 | 4.90E-04 |
| O-15 Oxygen gas, continuous inhalation | 4.0E-04 | 3.67E-04 | -8 | **2.17E-04** | -46 | 2.14E-04 | 2.21E-04 |
| O-15 Oxygen gas, single inhalation | 3.7E-04 | 3.33E-04 | -10 | **1.96E-04** | -47 | 1.91E-04 | 2.00E-04 |
| O-15 Oxygen Monoxide, continuous inhalation for 1 hr | 5.5E-04 | 5.51E-04 | 45 | **4.24E-04** | 12 | 5.35E-04 | 3.13E-04 |
| O-15 Oxygen Monoxide, single inhalation with 20 s breathhold | 8.1E-04 | 8.11E-04 | 59 | **6.30E-04** | 24 | 8.00E-04 | 4.59E-04 |
| O-15 Oxygen water | 1.1E-03 | 9.07E-04 | -18 | **8.29E-04** | -25 | 8.30E-04 | 8.29E-04 |
| P-32 Phosphate | 2.4E+00 | 7.49E-01 | -69 | **8.58E-01** | -64 | 8.54E-01 | 8.63E-01 |
| P-33 Phosphate | 6.6E-01 | 1.08E-01 | -84 | **1.23E-01** | -81 | 1.22E-01 | 1.25E-01 |
| Rb-81 Rubidium | 2.8E-02 | 1.60E-02 | -43 | **1.79E-02** | -36 | 1.78E-02 | 1.80E-02 |
| Rb-81 Rubidium-labelled Denatured erythrocytes | 1.4E-01 | 1.07E-01 | -24 | **5.44E-02** | -61 | 4.99E-02 | 5.89E-02 |
| Rb-82 Rubidium ultrashort lived | 3.4E-03 | 3.41E-03 | 0 | **3.05E-03** | -10 | 2.78E-03 | 3.33E-03 |
| Rb-84 Rubidium | 2.8E+00 | 2.65E+00 | -5 | **2.96E+00** | 6 | 2.97E+00 | 2.95E+00 |
| Rb-86 Rubidium | 3.0E+00 | 8.15E-01 | -73 | **9.50E-01** | -68 | 9.47E-01 | 9.53E-01 |
| S-35 Sulfur Sulphate | 9.0E-02 | 8.75E-02 | -3 | **9.98E-02** | 11 | 9.93E-02 | 1.00E-01 |
| Sc-46 Scandium labelled non-absorbable markers fluids | 1.6E+00 | 7.73E-01 | -52 | **8.00E-01** | -50 | 6.83E-01 | 9.18E-01 |
| Sc-46 Scandium labelled non-absorbable markers solids | 1.7E+00 | 8.02E-01 | -53 | **8.31E-01** | -51 | 7.06E-01 | 9.56E-01 |
| Sc-47 Scandium labelled non-absorbable markers fluids | 7.4E-01 | 3.83E-02 | -95 | **3.97E-02** | -95 | 3.52E-02 | 4.42E-02 |
| Sc-47 Scandium labelled non-absorbable markers solids | 7.6E-01 | 3.97E-02 | -95 | **4.12E-02** | -95 | 3.63E-02 | 4.60E-02 |
| Se-75 Selenium 1-Selenomethionine | 2.5E+00 | 2.42E+00 | -3 | **2.68E+00** | 7 | 2.73E+00 | 2.64E+00 |
| Se-75 Selenium labelled amino acids | 2.2E+00 | 2.03E+00 | -8 | **2.21E+00** | 0 | 2.33E+00 | 2.09E+00 |
| Se-75 Selenium labelled bibe acid SeHCAT | 6.9E-01 | 2.37E-01 | -66 | **2.77E-01** | -60 | 2.76E-01 | 2.77E-01 |
| Se-75 Selenium Selenite | 2.6E+00 | 2.80E+00 | 8 | **2.99E+00** | 15 | 2.90E+00 | 3.08E+00 |
| Se-75 Selenium Selenomethylcholesterol | 1.5E+00 | 1.38E+00 | -8 | **1.48E+00** | -1 | 1.52E+00 | 1.44E+00 |
| Sr-85 Strontium | 7.9E-01 | 5.44E-01 | -31 | **6.14E-01** | -22 | 6.10E-01 | 6.17E-01 |
| Sr-87m Strontium | 6.4E-03 | 5.03E-03 | -21 | **5.73E-03** | -10 | 5.87E-03 | 5.60E-03 |
| Sr-89 Strontium | 3.1E+00 | 8.36E-01 | -73 | **9.45E-01** | -70 | 9.33E-01 | 9.58E-01 |
| Tc-99m Technetium Apcitide | 4.7E-03 | 1.90E-03 | -60 | **2.05E-03** | -56 | 2.01E-03 | 2.09E-03 |
| Tc-99m Technetium DMSA | 8.8E-03 | 1.44E-02 | 64 | **1.32E-02** | 50 | 1.27E-02 | 1.38E-02 |
| Tc-99m Technetium DTPA, (intrathecal administered) cisternal injection | 4.7E-03 | 4.04E-03 | -14 | **4.57E-03** | -3 | 4.78E-03 | 4.36E-03 |
| Tc-99m Technetium DTPA, (intrathecal administered) lumbar injection | 4.8E-03 | 3.96E-03 | -18 | **4.48E-03** | -7 | 4.74E-03 | 4.23E-03 |
| Tc-99m Technetium DTPA, abnormal renal function | 4.6E-03 | 4.01E-03 | -13 | **4.46E-03** | -3 | 4.71E-03 | 4.21E-03 |
| Tc-99m Technetium DTPA, normal renal function | 4.9E-03 | 3.28E-03 | -33 | **3.72E-03** | -24 | 4.33E-03 | 3.12E-03 |
| Tc-99m Technetium EC, abnormal renal function | 4.6E-03 | 3.71E-03 | -19 | **4.08E-03** | -11 | 4.49E-03 | 3.68E-03 |
| Tc-99m Technetium EC, acute renal function | 9.9E-03 | 1.55E-02 | 57 | **1.41E-02** | 42 | 1.37E-02 | 1.45E-02 |
| Tc-99m Technetium EC, normal renal function | 6.3E-03 | 3.69E-03 | -41 | **4.23E-03** | -33 | 5.12E-03 | 3.33E-03 |
| Tc-99m Technetium ECD | 7.7E-03 | 5.36E-03 | -30 | **5.75E-03** | -25 | 6.13E-03 | 5.36E-03 |
| Tc-99m Technetium furifosmin, exercise | 8.9E-03 | 6.25E-03 | -30 | **6.67E-03** | -25 | 6.73E-03 | 6.60E-03 |
| Tc-99m Technetium furifosmin, resting subject | 1.0E-02 | 6.53E-03 | -35 | **6.99E-03** | -30 | 7.07E-03 | 6.91E-03 |
| Tc-99m Technetium gluconate glucoheptonate | 5.4E-03 | 6.35E-03 | 18 | **6.42E-03** | 19 | 6.77E-03 | 6.07E-03 |
| Tc-99m Technetium labelled aerosols Substances with fast clearance from lungs | 6.1E-03 | 5.03E-03 | -18 | **4.52E-03** | -26 | 4.73E-03 | 4.31E-03 |
| Tc-99m Technetium labelled aerosols Substances with slow clearance from lungs | 1.4E-02 | 1.65E-02 | 18 | **1.22E-02** | -13 | 1.12E-02 | 1.31E-02 |
| Tc-99m Technetium labelled HSA | 6.1E-03 | 1.53E-03 | -75 | **1.65E-03** | -73 | 1.62E-03 | 1.68E-03 |
| Tc-99m Technetium labelled albumin, (intrathecal administered) cisternal injection | 5.0E-03 | 2.33E-04 | -95 | **2.52E-04** | -95 | 2.47E-04 | 2.56E-04 |
| Tc-99m Technetium labelled albumin, (intrathecal administered) Lumbar injection | 5.2E-03 | 3.69E-04 | -93 | **4.00E-04** | -92 | 3.93E-04 | 4.07E-04 |
| Tc-99m Technetium labelled albumin microspheres | 1.0E-02 | 1.16E-02 | 16 | **9.19E-03** | -8 | 8.72E-03 | 9.66E-03 |
| Tc-99m Technetium labelled citrate complex | 6.1E-03 | 6.44E-03 | 6 | **6.52E-03** | 7 | 6.74E-03 | 6.31E-03 |
| Tc-99m Technetium labelled colloids, large colloids early to intermediate diffuse parenchymal liver disease | 1.1E-02 | 1.01E-02 | -8 | **8.14E-03** | -26 | 7.79E-03 | 8.48E-03 |
| Tc-99m Technetium labelled colloids, large colloids intermediate to advance diffuse parenchymal liver disease | 1.3E-02 | 1.30E-02 | 0 | **9.20E-03** | -29 | 8.70E-03 | 9.70E-03 |
| Tc-99m Technetium labelled colloids large colloids normal liver condition | 9.4E-03 | 7.37E-03 | -22 | **7.24E-03** | -23 | 7.04E-03 | 7.44E-03 |
| Tc-99m Technetium labelled colloids, small colloids early to intermediate diffuse parenchymal liver disease | 1.2E-02 | 1.04E-02 | -13 | **8.46E-03** | -30 | 8.09E-03 | 8.84E-03 |
| Tc-99m Technetium labelled colloids, small colloids intermediate to advance diffuse parenchymal liver disease | 1.3E-02 | 1.32E-02 | 2 | **9.37E-03** | -28 | 8.85E-03 | 9.89E-03 |
| Tc-99m Technetium labelled colloids small colloids normal liver condition | 9.7E-03 | 1.46E-02 | 51 | **1.52E-02** | 57 | 1.48E-02 | 1.55E-02 |
| Tc-99m Technetium labelled denatured erythrocytes | 1.9E-02 | 2.59E-02 | 36 | **1.46E-02** | -23 | 1.36E-02 | 1.56E-02 |
| Tc-99m Technetium labelled erythrocytes | 7.0E-03 | 2.57E-03 | -63 | **2.69E-03** | -62 | 2.67E-03 | 2.71E-03 |
| Tc-99m Technetium labelled fibrinogen | 6.2E-03 | 1.68E-03 | -73 | **1.82E-03** | -71 | 1.79E-03 | 1.85E-03 |
| Tc-99m Technetium labelled Heparin | 5.5E-03 | 3.55E-03 | -35 | **3.99E-03** | -27 | 4.34E-03 | 3.64E-03 |
| Tc-99m Technetium labelled HM-PAO | 9.3E-03 | 1.06E-02 | 14 | **1.01E-02** | 9 | 9.93E-03 | 1.04E-02 |
| Tc-99m Technetium labelled human immunoglobulin (HIG) | 7.0E-03 | 4.72E-03 | -33 | **4.59E-03** | -34 | 4.89E-03 | 4.29E-03 |
| Tc-99m Technetium labelled IDA derivatives, normal hepato-biliary conditions | 1.7E-02 | 7.70E-03 | -55 | **8.62E-03** | -49 | 8.58E-03 | 8.66E-03 |
| Tc-99m Technetium labelled IDA derivatives, occlusion of the common bile duct | 7.5E-03 | 4.85E-03 | -35 | **6.17E-03** | -18 | 6.31E-03 | 6.04E-03 |
| Tc-99m Technetium labelled IDA derivatives, occlusion of the cystic duct | 1.4E-02 | 7.30E-03 | -48 | **8.06E-03** | -42 | 8.23E-03 | 7.89E-03 |
| Tc-99m Technetium labelled IDA, derivatives parenchymal liver disease | 9.3E-03 | 5.26E-03 | -43 | **5.98E-03** | -36 | 6.51E-03 | 5.45E-03 |
| Tc-99m Technetium labelled MAA | 1.1E-02 | 1.29E-02 | 17 | **1.02E-02** | -7 | 9.54E-03 | 1.08E-02 |
| Tc-99m Technetium labelled MAG3, abnormal renal function | 6.1E-03 | 4.31E-03 | -29 | **4.74E-03** | -22 | 5.49E-03 | 3.98E-03 |
| Tc-99m Technetium labelled MAG3, acute unilateral renal blockage abnormal kidney | 1.0E-02 | 1.54E-02 | 54 | **1.40E-02** | 40 | 1.38E-02 | 1.43E-02 |
| Tc-99m Technetium labelled MAG3, acute unilateral renal blockage normal kidney | 1.0E-02 | 4.12E-03 | -59 | **4.70E-03** | -53 | 5.30E-03 | 4.10E-03 |
| Tc-99m Technetium labelled MAG3,normal renal function | 7.0E-03 | 4.05E-03 | -42 | **4.65E-03** | -34 | 5.68E-03 | 3.62E-03 |
| Tc-99m Technetium labelled MIBI, exercise | 7.9E-03 | 6.06E-03 | -33 | **6.55E-03** | -27 | 6.57E-03 | 6.52E-03 |
| Tc-99m Technetium labelled MIBI, resting subject | 9.0E-03 | 6.58E-03 | -17 | **7.03E-03** | -11 | 6.95E-03 | 7.11E-03 |
| Tc-99m Technetium labelled monoclonal antibodies fab fragments | 1.7E-02 | 1.48E-02 | -13 | **1.46E-02** | -14 | 1.40E-02 | 1.51E-02 |
| Tc-99m Technetium labelled monoclonal antibodies fab2 fragments | 1.1E-02 | 1.18E-02 | 7 | **1.22E-02** | 11 | 1.18E-02 | 1.26E-02 |
| Tc-99m Technetium labelled monoclonal antibodies, intact antibody | 1.2E-02 | 8.27E-03 | -31 | **8.18E-03** | -32 | 7.95E-03 | 8.40E-03 |
| Tc-99m Technetium labelled non-absorbable markers, orally administered of fluids | 1.9E-02 | 9.88E-03 | -48 | **1.06E-02** | -44 | 1.04E-02 | 1.08E-02 |
| Tc-99m Technetium labelled non-absorbable markers, orally administered of solids | 2.4E-02 | 1.08E-02 | -55 | **1.14E-02** | -53 | 1.11E-02 | 1.18E-02 |
| Tc-99m Technetium labelled phosphates and phosphonates, high bone uptake and/or severely impaired kidney function | 4.7E-03 | 3.01E-03 | -36 | **3.49E-03** | -26 | 3.50E-03 | 3.49E-03 |
| Tc-99m Technetium labelled phosphates and phosphonates, normal uptake and excretion | 5.8E-03 | 3.80E-03 | -34 | **4.31E-03** | -26 | 4.86E-03 | 3.75E-03 |
| Tc-99m Technetium labelled Plasmin | 7.3E-03 | 6.07E-03 | -17 | **5.13E-03** | -30 | 4.98E-03 | 5.29E-03 |
| Tc-99m Technetium labelled platelets (thrombocytes) | 1.2E-02 | 1.26E-02 | 5 | **8.21E-03** | -32 | 7.79E-03 | 8.64E-03 |
| Tc-99m Technetium labelled small colloids, intratumoural adm time to removal 6 h | 1.2E-03 | 1.78E-03 | 48 | **2.24E-03** | 87 | 1.98E-03 | 2.50E-03 |
| Tc-99m Technetium labelled small colloids, intratumoural adm time to removal 18 h | 2.0E-03 | 3.14E-03 | 57 | **3.96E-03** | 98 | 3.49E-03 | 4.43E-03 |
| Tc-99m Technetium labelled tetrofosmin, exercise | 6.9E-03 | 5.18E-03 | -25 | **5.76E-03** | -17 | 5.86E-03 | 5.66E-03 |
| Tc-99m Technetium labelled tetrofosmin, resting subject | 8.0E-03 | 5.84E-03 | -27 | **6.29E-03** | -21 | 6.36E-03 | 6.22E-03 |
| Tc-99m Technetium labelled white blood cells (leukocytes) | 1.1E-02 | 9.60E-03 | -13 | **7.17E-03** | -35 | 6.81E-03 | 7.54E-03 |
| Tc-99m Technetium pertechnegas | 1.2E-02 | 1.46E-02 | 22 | **1.46E-02** | 22 | 1.41E-02 | 1.50E-02 |
| Tc-99m Technetium pertechnetate, intravenous blocking agent given | 4.2E-03 | 3.66E-03 | -13 | **4.12E-03** | -2 | 4.47E-03 | 3.78E-03 |
| Tc-99m Technetium pertechnetate, intravenous no blocking agent given | 1.3E-02 | 1.55E-02 | 19 | **1.59E-02** | 22 | 1.55E-02 | 1.64E-02 |
| Tc-99m Technetium pertechnetate, orally no blocking agent given | 1.4E-02 | 6.02E-03 | -57 | **6.38E-03** | -54 | 6.33E-03 | 6.43E-03 |
| Tc-99m Technetium technegas | 1.5E-02 | 1.87E-02 | 25 | **1.36E-02** | -9 | 1.24E-02 | 1.49E-02 |
| Tl-201 Thallium ion | 1.4E-01 | 1.21E-01 | -14 | **1.02E-01** | -27 | 1.07E-01 | 9.76E-02 |
| Xe-127 Xenon, rebreathing for 10 min | 1.1E-03 | 1.04E-03 | -5 | **1.17E-03** | 6 | 1.20E-03 | 1.14E-03 |
| Xe-127 Xenon, rebreathing for 5 min | 7.1E-04 | 6.67E-04 | -6 | **7.43E-04** | 5 | 7.60E-04 | 7.26E-04 |
| Xe-127 Xenon, single inhalation with 30 s breathhold or intravenous injection with 30 s breathhold | 1.3E-04 | 1.38E-04 | 6 | **1.37E-04** | 5 | 1.36E-04 | 1.38E-04 |
| Xe-133 Xenon, rebreathing for 10 min | 1.1E-03 | 1.12E-03 | 2 | **1.28E-03** | 16 | 1.27E-03 | 1.28E-03 |
| Xe-133 Xenon, rebreathing for 5 min | 7.3E-04 | 7.22E-04 | -1 | **7.93E-04** | 9 | 7.87E-04 | 7.99E-04 |
| Xe-133 Xenon, single inhalation with 30 s breathhold or intravenous injection with 30 s breathhold | 1.8E-04 | 1.84E-04 | 2 | **1.50E-04** | -17 | 1.45E-04 | 1.55E-04 |
| Yb-169 Ytterbium DTPA, (intrathecal administered) cisternal injection | 1.4E-01 | 1.36E-01 | -3 | **1.54E-01** | 10 | 1.59E-01 | 1.49E-01 |
| Yb-169 Ytterbium DTPA, (intrathecal administered) lumbar injection | 1.2E-01 | 1.17E-01 | -2 | **1.32E-01** | 10 | 1.37E-01 | 1.28E-01 |
| Yb-169 Ytterbium DTPA, abnormal renal function | 7.3E-02 | 7.11E-02 | -3 | **7.82E-02** | 7 | 8.10E-02 | 7.54E-02 |
| Yb-169 Ytterbium DTPA, normal renal function | 3.6E-02 | 2.16E-02 | -40 | **2.40E-02** | -33 | 2.68E-02 | 2.12E-02 |
| Zn-62 Zink | 3.5E-01 | 2.74E-02 | -92 | **3.44E-02** | -90 | 3.33E-02 | 3.54E-02 |
| Zn-65 Zink | 8.4E+00 | 8.52E+00 | 1 | **9.94E+00** | 18 | 9.90E+00 | 9.98E+00 |
| Zn-69m Zink | 1.4E-01 | 3.68E-02 | -74 | **4.68E-02** | -67 | 4.55E-02 | 4.80E-02 |
