# Supplementary figures and images for: Effective dose to adult patients from 338 radiopharmaceuticals estimated using ICRP biokinetic data, ICRP/ICRU computational reference phantoms and ICRP 2007 tissue weighting factors
Source: EJNMMI Phys. 2014 Sep 29;1:9. doi: 10.1186/2197-7364-1-9 (PMC4545621; doi:10.1186/2197-7364-1-9)

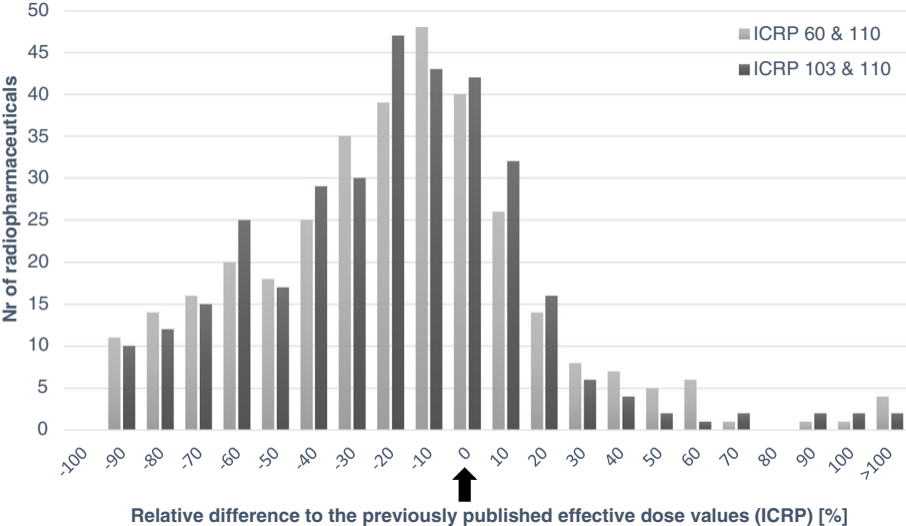

Supplement: Supplementary file 2 — Authors’ original file for figure 1 [file 40658_2014_116_MOESM2_ESM.pdf]
