# Supplementary material for: Erratum to: Effective dose to adult patients from 338 radiopharmaceuticals estimated using ICRP biokinetic data, ICRP/ICRU computational reference phantoms and ICRP 2007 tissue weighting factors
Source: EJNMMI Phys. 2015 Sep 30;2:22. doi: 10.1186/s40658-015-0121-4 (PMC4724792; doi:10.1186/s40658-015-0121-4)
Supplement: Additional file 1: Table S1. — Effective dose from all the radiopharmaceuticals published by the ICRP, determined using three different methods. (E/A0) 1 is the previously published effective dose per unit administered activity (E/A0) by ICRP, (E/A0)2 is (E/A0) dose calculated with the new phantoms and old tissue weighting factors while (E/A0) 3 is with the new phantoms and new weighting factors. (E/A0)2-(E/A0) 1))/ (E/A0)1 and ((E/A0) 3-(E/A0)1)/ (E/A0)1 is the difference in % of the new values compared to the old. (E/A0)3 male and (E/A0)3 are the effective dose estimations generated from the equivalent dose of each gender separately using the new phantoms and new weighting factors. (DOCX 65 kb) [file 40658_2015_121_MOESM1_ESM.docx]

**Table S1**. Effective dose from all the radiopharmaceuticals published by the ICRP, determined using three different methods. (E/A_0_) 1 is the previously published effective dose per unit administered activity (E/A_0_) by ICRP, (E/A_0_)2 is (E/A_0_) dose calculated with the new phantoms and old tissue weighting factors while (E/A_0_) 3 is with the new phantoms and new weighting factors. (E/A_0_)2-(E/A_0_) 1))/ (E/A_0_)1 and ((E/A_0_) 3-(E/A_0_)1)/ (E/A_0_)1 is the difference in % of the new values compared to the old. (E/A_0_)3 male and (E/A_0_)3 are the effective dose estimations generated from the equivalent dose of each gender separately using the new phantoms and new weighting factors.

| **Radiopharmaceuticals** | (E/A_0_)**1**  **[mSv/**  **MBq]** | (E/A_0_)**2**  **[mSv/**  **MBq]** | **(**(E/A_0_)**2-**(E/A_0_)**1) /**(E/A_0_)**1**  **[%]** | **(E/A_0_)3**  **[mSv/**  **MBq]** | **(**(E/A_0_)**3-**(E/A_0_)**1) /**(E/A_0_)**1**  **[%]** | (E/A_0_)**3 male**  **[mSv/**  **MBq]** | (E/A_0_)**3 female**  **[mSv/**  **MBq]** |
| --- | --- | --- | --- | --- | --- | --- | --- |
| **Phantom** | **MIRD** | **ICRP/**  **ICRU** |  | **ICRP/**  **ICRU** |  | **ICRP/**  **ICRU** | **ICRP/**  **ICRU** |
| **w_T_** | **ICRP**  **60** | **ICRP**  **60** |  | **ICRP 103** |  | **ICRP 103** | **ICRP 103** |
| As-72 Arsenate Arsenite | 3.60E-01 | 3.54E-01 | -2 | **3.52E-01** | -2 | 3.16E-01 | 3.87E-01 |
| As-74 Arsenate Arsenite | 5.10E-01 | 5.46E-01 | 7 | **5.27E-01** | 3 | 4.78E-01 | 5.75E-01 |
| As-76 Arsenate Arsenite | 2.80E-01 | 2.59E-01 | -8 | **2.51E-01** | -10 | 2.25E-01 | 2.76E-01 |
| Au-198 Gold colloid early to intermediate diffuse parenchymal liver disease | 1.40E+00 | 1.25E+00 | -11 | **7.87E-01** | -44 | 7.08E-01 | 8.65E-01 |
| Au-198 Gold colloid intermediate to advanced diffuse parenchymal liver disease | 1.70E+00 | 1.47E+00 | -14 | **8.21E-01** | -52 | 7.40E-01 | 9.02E-01 |
| Au-198 Gold colloid normal liver condition | 1.10E+00 | 1.06E+00 | -4 | **8.01E-01** | -27 | 7.22E-01 | 8.81E-01 |
| Ba-131 Barium | 5.00E-01 | 3.65E-01 | -27 | **3.48E-01** | -30 | 2.84E-01 | 4.13E-01 |
| Ba-131 Barium labelled non-absorbable markers orally administered of fluids | 4.90E-01 | 2.50E-01 | -49 | **2.30E-01** | -53 | 1.77E-01 | 2.82E-01 |
| Ba-131 Barium labelled non-absorbable markers orally administered of solids | 5.10E-01 | 2.58E-01 | -50 | **2.38E-01** | -53 | 1.83E-01 | 2.93E-01 |
| Ba-133m Barium | 4.70E-01 | 1.05E-01 | -78 | **1.05E-01** | -78 | 8.99E-02 | 1.20E-01 |
| Ba-135m Barium | 3.40E-01 | 7.87E-02 | -77 | **7.88E-02** | -77 | 6.83E-02 | 8.92E-02 |
| Br-76 Bromide | 2.80E-01 | 3.08E-01 | 10 | **3.06E-01** | 9 | 2.78E-01 | 3.34E-01 |
| Br-77 Bromide | 7.70E-02 | 8.08E-02 | 5 | **7.96E-02** | 3 | 7.25E-02 | 8.66E-02 |
| Br-77 Bromide Bromospiperone | 8.50E-02 | 1.01E-01 | 19 | **1.04E-01** | 22 | 9.35E-02 | 1.14E-01 |
| Br-82 Bromide | 4.00E-01 | 2.96E-02 | -93 | **3.01E-02** | -92 | 2.71E-02 | 3.30E-02 |
| C-11 Carbon (2-11C)thymidine | 2.70E-03 | 3.01E-03 | 11 | **3.04E-03** | 13 | 2.77E-03 | 3.32E-03 |
| C-11 Carbon (Methyl-11C)thymidine | 3.50E-03 | 4.11E-03 | 17 | **4.29E-03** | 23 | 3.91E-03 | 4.68E-03 |
| C-11 Carbon acetate | 3.50E-03 | 4.26E-03 | 22 | **3.65E-03** | 4 | 3.33E-03 | 3.97E-03 |
| C-11 Carbon amino acids | 5.60E-03 | 5.76E-03 | 3 | **5.26E-03** | -6 | 4.91E-03 | 5.61E-03 |
| C-11 Carbon brain receptor substances | 4.30E-03 | 3.70E-03 | -14 | **3.62E-03** | -16 | 3.23E-03 | 4.00E-03 |
| C-11 Carbon COHb-Labelled erythrocytes | 5.00E-03 | 8.26E-03 | 65 | **8.66E-03** | 73 | 8.00E-03 | 9.31E-03 |
| C-11 Carbon Dioxide continuous inhalation for 1 hr | 1.00E-03 | 1.07E-03 | 7 | **1.08E-03** | 8 | 9.77E-04 | 1.17E-03 |
| C-11 Carbon Dioxide single inhalation with 20 s breathhold | 1.60E-03 | 1.67E-03 | 4 | **1.68E-03** | 5 | 1.52E-03 | 1.83E-03 |
| C-11 Carbon Methionine | 8.40E-03 | 5.88E-03 | -30 | **5.11E-03** | -39 | 4.50E-03 | 5.72E-03 |
| C-11 Carbon Monoxide continuous inhalation for 1 hr | 3.20E-03 | 5.13E-03 | 60 | **5.37E-03** | 68 | 4.96E-03 | 5.78E-03 |
| C-11 Carbon Monoxide single inhale 20 s breathhold (2,5L) | 4.80E-03 | 7.80E-03 | 62 | **8.17E-03** | 70 | 7.55E-03 | 8.79E-03 |
| C-11 Carbon Realistic maximum | 1.10E-02 | 6.04E-03 | -45 | **5.08E-03** | -54 | 4.36E-03 | 5.80E-03 |
| C-11 Carbon Spiperone | 5.30E-03 | 5.99E-03 | 13 | **6.03E-03** | 14 | 5.44E-03 | 6.62E-03 |
| C-14 Carbon Inulin abnormal renal function | 1.50E-02 | 1.13E-02 | -25 | **1.09E-02** | -27 | 9.87E-03 | 1.20E-02 |
| C-14 Carbon Inulin normal renal function | 8.20E-03 | 1.27E-03 | -85 | **1.15E-03** | -86 | 1.04E-03 | 1.26E-03 |
| C-14 Carbon labelled neutral fat & free fatty acids | 2.10E+00 | 1.88E+00 | -10 | **2.65E+00** | 26 | 3.14E+00 | 2.15E+00 |
| C-14 Carbon labelled urea 14-C, helicobacter positive patient orally administered | 8.10E-02 | 8.62E-02 | 6 | **8.79E-02** | 9 | 7.96E-02 | 9.62E-02 |
| C-14 Carbon labelled urea 14-C, normal case orally administered | 3.10E-02 | 2.67E-02 | -14 | **2.72E-02** | -12 | 2.46E-02 | 2.98E-02 |
| Ca-45 Calcium, intravenous | 3.10E+00 | 1.54E-01 | -95 | **1.56E-01** | -95 | 1.43E-01 | 1.69E-01 |
| Ca-45 Calcium, orally | 1.80E+00 | 1.12E-01 | -94 | **1.14E-01** | -94 | 1.01E-01 | 1.26E-01 |
| Ca-47 Calcium, intravenous | 1.20E+00 | 5.12E-01 | -57 | **5.18E-01** | -57 | 4.62E-01 | 5.74E-01 |
| Ca-47 Calcium, orally | 1.80E+00 | 6.17E-01 | -66 | **6.08E-01** | -66 | 5.11E-01 | 7.04E-01 |
| Cl-34m Chloride (47% of Cl-34 decays) | 1.40E-02 | 1.14E-02 | -18 | **1.14E-02** | -18 | 1.04E-02 | 1.25E-02 |
| Cl-36 Chloride | 6.70E-01 | 8.27E-01 | 23 | **8.42E-01** | 26 | 7.61E-01 | 9.22E-01 |
| Cl-38 Chloride | 1.40E-02 | 1.55E-02 | 11 | **1.56E-02** | 11 | 1.41E-02 | 1.71E-02 |
| Co-57 Cobalt labelled bleomycin | 4.70E-02 | 1.97E-02 | -58 | **2.01E-02** | -57 | 1.85E-02 | 2.17E-02 |
| Co-57 Cobalt Vitamin B12, intravenous with carrier | 4.60E-01 | 6.23E-01 | 35 | **6.29E-01** | 37 | 5.86E-01 | 6.71E-01 |
| Co-57 Cobalt Vitamin B12, intravenous without carrier | 4.40E+00 | 6.30E+00 | 43 | **6.40E+00** | 45 | 5.99E+00 | 6.81E+00 |
| Co-57 Cobalt Vitamin B12, orally with flushing | 2.10E+00 | 2.94E+00 | 40 | **2.99E+00** | 42 | 2.80E+00 | 3.18E+00 |
| Co-57 Cobalt Vitamin B12, orally without flushing | 3.10E+00 | 4.41E+00 | 42 | **4.48E+00** | 45 | 4.19E+00 | 4.77E+00 |
| Co-58 Cobalt Vitamin B12, intravenous without carrier | 8.20E+00 | 1.22E+01 | 49 | **1.30E+01** | 59 | 1.22E+01 | 1.38E+01 |
| Co-58 Cobalt Vitamin B12, intravenous with carrier | 8.90E-01 | 1.22E+00 | 37 | **1.28E+00** | 44 | 1.20E+00 | 1.37E+00 |
| Co-58 Cobalt Vitamin B12, orally with flushing | 4.00E+00 | 5.77E+00 | 44 | **6.15E+00** | 54 | 5.76E+00 | 6.54E+00 |
| Co-58 Cobalt Vitamin B12, orally without flushing | 5.90E+00 | 8.61E+00 | 46 | **9.20E+00** | 56 | 8.61E+00 | 9.78E+00 |
| Cr-51 Chromium(III) Chloride | 6.80E-02 | 9.32E-02 | 37 | **9.94E-02** | 46 | 9.18E-02 | 1.07E-01 |
| Cr-51 Chromium EDTA, intravenous abnormal renal function | 4.60E-03 | 4.60E-03 | 0 | **4.44E-03** | -3 | 3.99E-03 | 4.88E-03 |
| Cr-51 Chromium EDTA, intravenous normal renal function | 2.00E-03 | 1.65E-03 | -18 | **1.43E-03** | -29 | 1.23E-03 | 1.62E-03 |
| Cr-51 Chromium EDTA, orally | 4.40E-02 | 1.65E-02 | -63 | **1.52E-02** | -65 | 1.17E-02 | 1.87E-02 |
| Cr-51 Chromium labelled denatured erythrocytes | 1.80E-01 | 2.46E-01 | 36 | **1.44E-01** | -20 | 1.31E-01 | 1.58E-01 |
| Cr-51 Chromium labelled erythrocytes | 1.70E-01 | 2.81E-01 | 65 | **2.65E-01** | 56 | 2.43E-01 | 2.87E-01 |
| Cr-51 Chromium labelled non-absorbable markers, orally administered of fluids | 4.30E-02 | 1.65E-02 | -62 | **1.52E-02** | -65 | 1.17E-02 | 1.87E-02 |
| Cr-51 Chromium labelled non-absorbable markers, orally administered of solids | 4.50E-02 | 1.70E-02 | -62 | **1.57E-02** | -65 | 1.21E-02 | 1.94E-02 |
| Cr-51 Chromium labelled platelets | 1.40E-01 | 1.76E-01 | 26 | **1.31E-01** | -6 | 1.18E-01 | 1.43E-01 |
| Cr-51 Chromium labelled white blood cells (leukocytes) | 1.20E-01 | 1.26E-01 | 5 | **9.16E-02** | -24 | 8.18E-02 | 1.01E-01 |
| Cs-129 Caesium | 4.90E-02 | 4.35E-02 | -11 | **4.12E-02** | -16 | 3.71E-02 | 4.52E-02 |
| Cs-130 Caesium | 3.40E-03 | 5.13E-04 | -85 | **4.87E-04** | -86 | 4.57E-04 | 5.18E-04 |
| Cs-131 Caesium | 5.00E-02 | 4.15E-02 | -17 | **3.87E-02** | -23 | 3.49E-02 | 4.26E-02 |
| Cs-134 Caesium | 2.50E-03 | 2.55E-03 | 2 | **2.44E-03** | -2 | 2.22E-03 | 2.65E-03 |
| Cs-134m Caesium | 6.70E-03 | 2.52E-03 | -62 | **2.42E-03** | -64 | 2.31E-03 | 2.52E-03 |
| Cu-64 Copper | 3.60E-02 | 4.66E-02 | 29 | **4.53E-02** | 26 | 4.11E-02 | 4.96E-02 |
| Cu-67 Copper | 1.50E-01 | 1.75E-01 | 17 | **1.68E-01** | 12 | 1.51E-01 | 1.84E-01 |
| F-18 Fluoride | 2.40E-02 | 1.41E-02 | -41 | **1.29E-02** | -46 | 1.12E-02 | 1.47E-02 |
| F-18 Fluoride L-dopa | 2.50E-02 | 1.75E-02 | -30 | **1.57E-02** | -37 | 1.37E-02 | 1.76E-02 |
| F-18 Fluoride FDG | 1.90E-02 | 1.69E-02 | -11 | **1.71E-02** | -10 | 1.53E-02 | 1.88E-02 |
| F-18 Fluoride labelled amino acids | 2.30E-02 | 2.27E-02 | -1 | **2.07E-02** | -10 | 1.92E-02 | 2.21E-02 |
| F-18 Fluoride labelled brain receptor substances | 2.80E-02 | 2.01E-02 | -28 | **2.02E-02** | -28 | 1.82E-02 | 2.22E-02 |
| Fe-52 Iron, intravenous | 1.10E+00 | 6.60E-02 | -94 | **6.84E-02** | -94 | 5.96E-02 | 7.73E-02 |
| Fe-52 Iron, orally | 7.10E-01 | 1.25E-01 | -82 | **1.25E-01** | -82 | 1.14E-01 | 1.35E-01 |
| Fe-55 Iron, intravenous | 4.00E+00 | 5.54E+00 | 39 | **5.15E+00** | 29 | 4.79E+00 | 5.51E+00 |
| Fe-55 Iron, orally | 4.20E-01 | 5.66E-01 | 35 | **5.27E-01** | 25 | 4.90E-01 | 5.64E-01 |
| Fe-59 Iron, intravenous | 1.00E+01 | 1.55E+01 | 55 | **1.60E+01** | 60 | 1.46E+01 | 1.73E+01 |
| Fe-59 Iron, orally | 2.00E+00 | 2.15E+00 | 8 | **2.19E+00** | 10 | 1.94E+00 | 2.44E+00 |
| Ga-66 Gallium citrate | 3.20E-01 | 2.53E-01 | -21 | **2.50E-01** | -22 | 2.26E-01 | 2.74E-01 |
| Ga-67 Gallium citrate | 1.00E-01 | 9.29E-02 | -7 | **9.08E-02** | -9 | 8.14E-02 | 1.00E-01 |
| Ga-68 Gallium citrate | 2.00E-02 | 1.90E-02 | -5 | **1.88E-02** | -6 | 1.70E-02 | 2.06E-02 |
| Ga-68 Gallium labelled EDTA | 4.00E-02 | 2.41E-02 | -40 | **2.14E-02** | -47 | 1.89E-02 | 2.40E-02 |
| Ga-72 Gallium citrate | 3.40E-01 | 2.95E-01 | -13 | **2.91E-01** | -14 | 2.62E-01 | 3.19E-01 |
| H-3 Tritium Inulin abnormal renal function | 1.70E-03 | 1.30E-03 | -24 | **1.26E-03** | -26 | 1.13E-03 | 1.38E-03 |
| H-3 Tritium Inulin normal renal function | 9.40E-04 | 1.42E-04 | -85 | **1.29E-04** | -86 | 1.16E-04 | 1.42E-04 |
| H-3 Tritium labelled neutral fat & free fatty acids | 2.20E-01 | 9.81E-02 | -55 | **1.80E-01** | -18 | 2.44E-01 | 1.16E-01 |
| H-3 Tritium water | 1.50E-02 | 1.48E-02 | -1 | **1.50E-02** | 0 | 1.53E-02 | 1.47E-02 |
| Hg-197 Mercury BMHP | 1.40E-01 | 1.90E-01 | 36 | **1.37E-01** | -2 | 1.26E-01 | 1.49E-01 |
| Hg-197 Mercury Chlormerodrin | 8.70E-02 | 9.77E-02 | 12 | **5.87E-02** | -33 | 5.39E-02 | 6.35E-02 |
| Hg-197 Mercury Dichloride | 1.40E-01 | 1.82E-01 | 30 | **1.05E-01** | -25 | 9.68E-02 | 1.14E-01 |
| Hg-203 Mercury Chlormerodrin | 1.10E+00 | 1.12E+00 | 1 | **7.98E-01** | -27 | 7.37E-01 | 8.60E-01 |
| I-123 Iodine BMIPP | 1.60E-02 | 1.70E-02 | 6 | **1.71E-02** | 7 | 1.56E-02 | 1.87E-02 |
| I-123 Iodine HIPPURAN abnormal renal function | 9.80E-03 | 8.77E-03 | -11 | **7.99E-03** | -18 | 7.03E-03 | 8.94E-03 |
| I-123 Iodine HIPPURAN normal renal function | 1.20E-02 | 8.88E-03 | -26 | **7.06E-03** | -41 | 5.98E-03 | 8.15E-03 |
| I-123 Iodine HIPPURAN Unilateral renal blockage abnormal kidney | 3.00E-02 | 3.41E-02 | 14 | **2.17E-02** | -28 | 1.95E-02 | 2.40E-02 |
| I-123 Iodine HIPPURAN Unilateral renal blockage normal kidney | 1.30E-02 | 1.26E-02 | -3 | **1.15E-02** | -12 | 1.02E-02 | 1.27E-02 |
| I-123 Iodine IMP Bound iodine | 2.70E-02 | 3.09E-02 | 14 | **3.22E-02** | 19 | 2.93E-02 | 3.51E-02 |
| I-123 Iodine IMP Released iodine | 2.80E-02 | 3.19E-02 | 14 | **3.30E-02** | 18 | 3.00E-02 | 3.61E-02 |
| I-123 Iodine IPPA | 1.60E-02 | 1.72E-02 | 7 | **1.72E-02** | 8 | 1.56E-02 | 1.87E-02 |
| I-123 Iodine labelled Albumin HSA | 2.00E-02 | 3.18E-02 | 59 | **3.30E-02** | 65 | 3.02E-02 | 3.58E-02 |
| I-123 Iodine labelled albumin (intrathecal administered) cisternal injection | 1.80E-02 | 2.16E-02 | 20 | **2.16E-02** | 20 | 1.97E-02 | 2.35E-02 |
| I-123 Iodine labelled albumin (intrathecal administered) lumbar injection | 1.80E-02 | 2.29E-02 | 27 | **2.29E-02** | 27 | 2.09E-02 | 2.49E-02 |
| I-123 Iodine labelled brain receptor sub | 5.00E-02 | 3.60E-02 | -28 | **3.65E-02** | -27 | 3.30E-02 | 4.00E-02 |
| I-123 Iodine labelled fibrinogen bound iodine | 2.00E-02 | 3.28E-02 | 64 | **3.43E-02** | 72 | 3.14E-02 | 3.71E-02 |
| I-123 Iodine labelled fibrinogen released iodine | 1.30E-03 | 1.20E-03 | -8 | **1.10E-03** | -15 | 9.80E-04 | 1.22E-03 |
| I-123 Iodine labelled MAA, early to intermediate diffuse parenchymal liver disease | 1.90E-02 | 2.30E-02 | 21 | **1.90E-02** | 0 | 1.72E-02 | 2.08E-02 |
| I-123 Iodine labelled MAA, intermediate to advanced diffuse parenchymal liver disease | 2.20E-02 | 2.54E-02 | 15 | **1.94E-02** | -12 | 1.74E-02 | 2.14E-02 |
| I-123 Iodine labelled MAA, normal condition | 1.80E-02 | 2.13E-02 | 18 | **1.90E-02** | 6 | 1.73E-02 | 2.07E-02 |
| I-123 Iodine labelled monoclonal antibodies Fab fragments | 2.40E-02 | 1.90E-02 | -21 | **1.56E-02** | -35 | 1.41E-02 | 1.71E-02 |
| I-123 Iodine labelled monoclonal antibodies Fab2 fragments | 2.50E-02 | 2.19E-02 | -13 | **2.01E-02** | -20 | 1.82E-02 | 2.19E-02 |
| I-123 Iodine labelled monoclonal antibodies intact antibody | 2.90E-02 | 3.29E-02 | 13 | **2.94E-02** | 1 | 2.68E-02 | 3.21E-02 |
| I-123 Iodine MIBG | 1.30E-02 | 1.67E-02 | 28 | **1.67E-02** | 28 | 1.51E-02 | 1.82E-02 |
| I-123 Iodine Sodium rose Bengal, normal hepato-biliary condition | 5.90E-02 | 3.42E-02 | -42 | **3.38E-02** | -43 | 3.01E-02 | 3.75E-02 |
| I-123 Iodine Sodium rose Bengal, occlusion of the common bile duct | 2.50E-02 | 3.75E-02 | 50 | **3.79E-02** | 52 | 3.53E-02 | 4.05E-02 |
| I-123 Iodine Sodium rose Bengal, occlusion of the cystic duct | 4.50E-02 | 2.79E-02 | -38 | **2.68E-02** | -40 | 2.38E-02 | 2.98E-02 |
| I-123 Iodine Sodium rose Bengal, parenchymal liver disease | 2.70E-02 | 1.90E-02 | -30 | **1.78E-02** | -34 | 1.58E-02 | 1.99E-02 |
| I-123 Iodide Thyroid block uptake 0% | 1.10E-02 | 1.00E-02 | -9 | **9.23E-03** | -16 | 8.22E-03 | 1.02E-02 |
| I-123 Iodide Thyroid uptake 5% | 4.70E-02 | 4.86E-02 | 3 | **4.17E-02** | -11 | 3.82E-02 | 4.51E-02 |
| I-123 Iodide Thyroid uptake 15% | 1.10E-01 | 1.19E-01 | 8 | **9.90E-02** | -10 | 9.09E-02 | 1.07E-01 |
| I-123 Iodide Thyroid uptake 25% | 1.70E-01 | 1.89E-01 | 11 | **1.56E-01** | -8 | 1.43E-01 | 1.68E-01 |
| I-123 Iodide Thyroid uptake 35% | 2.20E-01 | 2.59E-01 | 18 | **2.13E-01** | -3 | 1.95E-01 | 2.30E-01 |
| I-123 Iodide Thyroid uptake 45% | 2.80E-01 | 3.29E-01 | 18 | **2.70E-01** | -4 | 2.48E-01 | 2.92E-01 |
| I-123 Iodide Thyroid uptake 55% | 3.40E-01 | 4.00E-01 | 18 | **3.27E-01** | -4 | 3.00E-01 | 3.53E-01 |
| I-124 Iodide Thyroid block uptake 0% | 9.50E-02 | 8.75E-02 | -8 | **8.08E-02** | -15 | 7.19E-02 | 8.97E-02 |
| I-124 Iodide Thyroid uptake 5% | 2.20E+00 | 2.11E+00 | -4 | **1.73E+00** | -21 | 1.58E+00 | 1.88E+00 |
| I-124 Iodide Thyroid uptake 15% | 6.40E+00 | 6.10E+00 | -5 | **4.98E+00** | -22 | 4.56E+00 | 5.41E+00 |
| I-124 Iodide Thyroid uptake 25% | 1.10E+01 | 1.01E+01 | -8 | **8.25E+00** | -25 | 7.55E+00 | 8.96E+00 |
| I-124 Iodide Thyroid uptake 35% | 1.50E+01 | 1.41E+01 | -6 | **1.15E+01** | -23 | 1.05E+01 | 1.25E+01 |
| I-124 Iodide Thyroid uptake 45% | 1.90E+01 | 1.81E+01 | -5 | **1.47E+01** | -23 | 1.35E+01 | 1.60E+01 |
| I-124 Iodide Thyroid uptake 55% | 2.30E+01 | 2.21E+01 | -4 | **1.80E+01** | -22 | 1.65E+01 | 1.95E+01 |
| I-125 Iodine Diiodothyronine | 3.60E-02 | 2.28E-02 | -37 | **2.12E-02** | -41 | 1.71E-02 | 2.53E-02 |
| I-125 Iodine HIPPURAN abnormal renal function | 7.00E-03 | 6.28E-03 | -10 | **5.76E-03** | -18 | 5.13E-03 | 6.38E-03 |
| I-125 Iodine HIPPURAN normal renal function | 7.70E-03 | 5.57E-03 | -28 | **4.51E-03** | -41 | 3.99E-03 | 5.04E-03 |
| I-125 Iodine HIPPURAN Unilateral renal blockage Abnormal kidney | 1.30E-01 | 1.70E-01 | 31 | **8.76E-02** | -33 | 7.96E-02 | 9.56E-02 |
| I-125 Iodine HIPPURAN Unilateral renal blockage normal kidney | 3.60E-02 | 4.14E-02 | 15 | **3.97E-02** | 10 | 3.56E-02 | 4.37E-02 |
| I-125 Iodine iodiantipyrine | 1.00E-02 | 9.91E-03 | -1 | **9.19E-03** | -8 | 8.22E-03 | 1.02E-02 |
| I-125 Iodine iodinated PVP | 6.50E-01 | 1.03E+00 | 59 | **1.03E+00** | 58 | 9.28E-01 | 1.13E+00 |
| I-125 Iodine iothalamate abnormal renal function | 1.40E-02 | 1.51E-02 | 8 | **1.45E-02** | 4 | 1.30E-02 | 1.60E-02 |
| I-125 Iodine iothalamate normal renal function | 7.20E-03 | 6.02E-03 | -16 | **5.23E-03** | -27 | 4.65E-03 | 5.81E-03 |
| I-125 iodine labelled Albumin HSA | 2.20E-01 | 3.91E-01 | 78 | **3.98E-01** | 81 | 3.59E-01 | 4.37E-01 |
| I-125 iodine labelled fibrinogen bound iodine | 8.00E-02 | 1.40E-01 | 74 | **1.44E-01** | 80 | 1.30E-01 | 1.58E-01 |
| I-125 iodine labelled fibrinogen released iodine | 8.00E-03 | 7.90E-03 | -1 | **7.26E-03** | -9 | 6.50E-03 | 8.03E-03 |
| I-125 Iodine labelled non-absorbable markers, orally administered of fluids | 1.70E-01 | 6.71E-02 | -61 | **6.20E-02** | -64 | 4.71E-02 | 7.69E-02 |
| I-125 Iodine labelled non-absorbable markers, orally administered of solids | 1.70E-01 | 6.93E-02 | -59 | **6.44E-02** | -62 | 4.89E-02 | 8.00E-02 |
| I-125 Iodine Reverse Triiodothyronine (rT3) | 3.70E-02 | 2.35E-02 | -37 | **2.19E-02** | -41 | 1.77E-02 | 2.61E-02 |
| I-125 Iodide Thyroid block uptake 0% | 9.10E-03 | 8.66E-03 | -5 | **7.94E-03** | -13 | 7.10E-03 | 8.78E-03 |
| I-125 Iodide Thyroid uptake 5% | 2.10E+00 | 2.66E+00 | 26 | **2.15E+00** | 2 | 1.98E+00 | 2.33E+00 |
| I-125 Iodide Thyroid uptake 15% | 6.20E+00 | 7.96E+00 | 28 | **6.45E+00** | 4 | 5.92E+00 | 6.98E+00 |
| I-125 Iodide Thyroid uptake 25% | 1.00E+01 | 1.33E+01 | 33 | **1.07E+01** | 7 | 9.85E+00 | 1.16E+01 |
| I-125 Iodide Thyroid uptake 35% | 1.40E+01 | 1.85E+01 | 32 | **1.50E+01** | 7 | 1.38E+01 | 1.62E+01 |
| I-125 Iodide Thyroid uptake 45% | 1.90E+01 | 2.39E+01 | 26 | **1.93E+01** | 2 | 1.78E+01 | 2.09E+01 |
| I-125 Iodide Thyroid uptake 55% | 2.30E+01 | 2.92E+01 | 27 | **2.36E+01** | 3 | 2.17E+01 | 2.55E+01 |
| I-125 Iodine Thyroxine | 1.00E-01 | 1.08E-01 | 8 | **1.04E-01** | 4 | 9.22E-02 | 1.17E-01 |
| I-125 Iodine Triiodothyronine (T3) | 4.70E-02 | 3.75E-02 | -20 | **3.56E-02** | -24 | 3.01E-02 | 4.11E-02 |
| I-131 Iodine Diiodothyronine | 2.50E-01 | 9.08E-02 | -64 | **8.70E-02** | -65 | 7.24E-02 | 1.02E-01 |
| I-131 Iodine HIPPURAN, abnormal renal function | 4.80E-02 | 3.22E-02 | -33 | **2.77E-02** | -42 | 2.44E-02 | 3.09E-02 |
| I-131 Iodine HIPPURAN, normal renal function | 5.20E-02 | 1.89E-02 | -64 | **1.53E-02** | -71 | 1.29E-02 | 1.78E-02 |
| I-131 Iodine HIPPURAN, unilateral renal blockage abnormal kidney | 6.60E-01 | 6.92E-01 | 5 | **3.50E-01** | -47 | 3.20E-01 | 3.79E-01 |
| I-131 Iodine HIPPURAN, unilateral renal blockage normal kidney | 1.80E-01 | 1.72E-01 | -4 | **1.69E-01** | -6 | 1.53E-01 | 1.85E-01 |
| I-131 Iodine Iodiantipyrine | 6.70E-02 | 5.10E-02 | -24 | **4.92E-02** | -27 | 4.41E-02 | 5.43E-02 |
| I-131 Iodine iodinated PVP | 6.00E-01 | 8.11E-01 | 35 | **8.06E-01** | 34 | 7.36E-01 | 8.76E-01 |
| I-131 Iodine labelled Albumin HSA | 6.40E-01 | 9.84E-01 | 54 | **1.01E+00** | 58 | 9.34E-01 | 1.09E+00 |
| I-131 Iodine labelled albumin (intrathecal administered) cisternal injection | 7.20E-01 | 9.45E-01 | 31 | **9.71E-01** | 35 | 8.95E-01 | 1.05E+00 |
| I-131 Iodine labelled albumin (intrathecal administered) lumbar injection | 3.00E-01 | 5.03E-01 | 68 | **5.29E-01** | 76 | 4.93E-01 | 5.65E-01 |
| I-131 Iodine labelled fibrinogen bound iodine | 4.20E-01 | 6.24E-01 | 49 | **6.48E-01** | 54 | 6.00E-01 | 6.96E-01 |
| I-131 Iodine labelled fibrinogen released iodine | 4.00E-02 | 2.90E-02 | -28 | **2.78E-02** | -31 | 2.48E-02 | 3.07E-02 |
| I-131 Iodine labelled MAA | 4.50E-01 | 4.91E-01 | 9 | **4.89E-01** | 9 | 4.39E-01 | 5.39E-01 |
| I-131 Iodine labelled microaggregated albumin early to intermediate diffuse parenchymal liver disease | 2.40E-01 | 2.65E-01 | 10 | **1.90E-01** | -21 | 1.72E-01 | 2.08E-01 |
| I-131 Iodine labelled microaggregated albumin intermediate to advanced diffuse parenchymal liver disease | 2.90E-01 | 3.01E-01 | 4 | **1.94E-01** | -33 | 1.76E-01 | 2.13E-01 |
| I-131 Iodine labelled microaggregated albumin normal condition | 2.20E-01 | 2.28E-01 | 4 | **1.86E-01** | -15 | 1.69E-01 | 2.03E-01 |
| I-131 Iodine labelled monoclonal antibodies, Fab fragments | 1.70E-01 | 1.03E-01 | -39 | **8.06E-02** | -53 | 7.28E-02 | 8.84E-02 |
| I-131 Iodine labelled monoclonal antibodies, Fab2 fragments | 2.00E-01 | 1.38E-01 | -31 | **1.21E-01** | -39 | 1.10E-01 | 1.33E-01 |
| I-131 Iodine labelled monoclonal antibodies, intact antibody | 4.70E-01 | 4.40E-01 | -6 | **3.59E-01** | -24 | 3.26E-01 | 3.94E-01 |
| I-131 Iodine labelled non-absorbable markers, orally administered of fluids | 1.20E+00 | 1.79E-01 | -85 | **1.65E-01** | -86 | 1.28E-01 | 2.02E-01 |
| I-131 Iodine labelled non-absorbable markers, orally administered of solids | 1.20E+00 | 1.84E-01 | -85 | **1.71E-01** | -86 | 1.33E-01 | 2.09E-01 |
| I-131 Iodine MIGB | 1.40E-01 | 1.50E-01 | 7 | **1.56E-01** | 11 | 1.41E-01 | 1.71E-01 |
| I-131 Iodine NP-59 | 1.80E+00 | 2.02E+00 | 12 | **1.74E+00** | -3 | 1.60E+00 | 1.89E+00 |
| I-131 Iodine Reverse Triiodothyronine (rT3) | 2.50E-01 | 9.52E-02 | -62 | **9.11E-02** | -64 | 7.61E-02 | 1.06E-01 |
| I-131 Iodine Sodium rose Bengal, normal hepato-biliary condition | 1.10E+00 | 1.55E-01 | -86 | **1.59E-01** | -86 | 1.58E-01 | 1.60E-01 |
| I-131 Iodine Sodium rose Bengal, occlusion of the common bile duct | 6.40E-01 | 8.64E-01 | 35 | **8.17E-01** | 28 | 7.47E-01 | 8.88E-01 |
| I-131 Iodine Sodium rose Bengal, occlusion of the cystic duct | 5.50E-01 | 1.40E-01 | -75 | **1.28E-01** | -77 | 1.01E-01 | 1.56E-01 |
| I-131 Iodine Sodium rose Bengal, parenchymal liver disease | 3.00E-01 | 8.90E-02 | -70 | **8.21E-02** | -73 | 6.65E-02 | 9.77E-02 |
| I-131 Iodide Thyroid block uptake 0% | 6.10E-02 | 4.34E-02 | -29 | **4.16E-02** | -32 | 3.71E-02 | 4.60E-02 |
| I-131 Iodide Thyroid uptake 5% | 3.60E+00 | 3.85E+00 | 7 | **3.11E+00** | -14 | 2.85E+00 | 3.36E+00 |
| I-131 Iodide Thyroid uptake 15% | 1.10E+01 | 1.15E+01 | 5 | **9.27E+00** | -16 | 8.51E+00 | 1.00E+01 |
| I-131 Iodide Thyroid uptake 25% | 1.70E+01 | 1.92E+01 | 13 | **1.54E+01** | -9 | 1.41E+01 | 1.66E+01 |
| I-131 Iodide Thyroid uptake 35% | 2.40E+01 | 2.68E+01 | 11 | **2.15E+01** | -10 | 1.98E+01 | 2.33E+01 |
| I-131 Iodide Thyroid uptake 45% | 3.10E+01 | 3.44E+01 | 11 | **2.77E+01** | -11 | 2.55E+01 | 3.00E+01 |
| I-131 Iodide Thyroid uptake 55% | 3.80E+01 | 4.21E+01 | 11 | **3.38E+01** | -11 | 3.11E+01 | 3.66E+01 |
| I-131 Iodine Thyroxine | 4.40E-01 | 4.04E-01 | -8 | **4.01E-01** | -9 | 3.61E-01 | 4.42E-01 |
| I-131 Iodine Triiodothyronine (T3) | 3.00E-01 | 1.69E-01 | -44 | **1.65E-01** | -45 | 1.44E-01 | 1.86E-01 |
| In-111 Indium | 2.10E-01 | 2.17E-01 | 3 | **2.09E-01** | 0 | 1.88E-01 | 2.31E-01 |
| In-111 Indium DTPA, abnormal renal function | 4.20E-02 | 4.37E-02 | 4 | **4.16E-02** | -1 | 3.75E-02 | 4.58E-02 |
| In-111 Indium DTPA, (intrathecal administered) cisternal injection | 6.30E-02 | 6.59E-02 | 5 | **6.29E-02** | 0 | 5.69E-02 | 6.89E-02 |
| In-111 Indium DTPA, (intrathecal administered) lumbar injection | 5.50E-02 | 5.63E-02 | 2 | **5.33E-02** | -3 | 4.81E-02 | 5.85E-02 |
| In-111 Indium DTPA, normal renal function | 2.10E-02 | 1.85E-02 | -12 | **1.55E-02** | -26 | 1.34E-02 | 1.77E-02 |
| In-111 Indium labelled aerosols substance with fast clearance from lungs | 2.50E-02 | 2.19E-02 | -13 | **1.97E-02** | -21 | 1.75E-02 | 2.20E-02 |
| In-111 Indium labelled aerosols substance with slow clearance from lungs | 2.40E-01 | 3.21E-01 | 34 | **3.47E-01** | 45 | 3.12E-01 | 3.83E-01 |
| In-111 Indium labelled bleomycin | 1.00E-01 | 1.18E-01 | 18 | **1.10E-01** | 10 | 1.00E-01 | 1.21E-01 |
| In-111 Indium labelled HIG | 1.70E-01 | 2.23E-01 | 31 | **2.15E-01** | 26 | 1.99E-01 | 2.31E-01 |
| In-111 Indium labelled monoclonal antibodies, Fab fragments | 3.70E-01 | 2.28E-01 | -38 | **2.17E-01** | -41 | 1.94E-01 | 2.39E-01 |
| In-111 Indium labelled monoclonal antibodies, Fab2 fragments | 3.60E-01 | 2.34E-01 | -35 | **2.26E-01** | -37 | 2.04E-01 | 2.48E-01 |
| In-111 Indium labelled monoclonal antibodies, intact antibody | 3.30E-01 | 2.88E-01 | -13 | **2.74E-01** | -17 | 2.49E-01 | 2.99E-01 |
| In-111 Indium labelled non-absorbable markers, orally administered of fluids | 3.10E-01 | 1.70E-01 | -45 | **1.59E-01** | -49 | 1.28E-01 | 1.90E-01 |
| In-111 Indium labelled non-absorbable markers, orally administered of solids | 3.20E-01 | 1.75E-01 | -45 | **1.65E-01** | -48 | 1.33E-01 | 1.97E-01 |
| In-111 Indium labelled plateletes (thrombocytes) | 3.90E-01 | 5.58E-01 | 43 | **4.36E-01** | 12 | 3.96E-01 | 4.76E-01 |
| In-111 Indium labelled white blood cells (leukocytes) | 3.60E-01 | 4.38E-01 | 22 | **3.45E-01** | -4 | 3.09E-01 | 3.80E-01 |
| In-111 Indium octreotide | 5.40E-02 | 6.74E-02 | 25 | **5.93E-02** | 10 | 5.34E-02 | 6.51E-02 |
| In-113m Indium | 1.00E-02 | 6.94E-03 | -31 | **6.22E-03** | -38 | 5.57E-03 | 6.87E-03 |
| In-113m Indium (colloidal) early to intermediate parenchymal liver disease | 1.40E-02 | 1.55E-02 | 11 | **1.05E-02** | -25 | 9.49E-03 | 1.15E-02 |
| In-113m Indium (colloidal) intermediate to advanced diffuse parenchymal liver disease | 1.80E-02 | 1.80E-02 | 0 | **1.08E-02** | -40 | 9.69E-03 | 1.18E-02 |
| In-113m Indium (colloidal) normal liver condition | 1.10E-02 | 1.29E-02 | 17 | **1.02E-02** | -7 | 9.20E-03 | 1.11E-02 |
| In-113m Indium DTPA, abnormal renal function | 5.50E-03 | 5.38E-03 | -2 | **5.13E-03** | -7 | 4.64E-03 | 5.63E-03 |
| In-113m Indium DTPA, normal renal function | 1.10E-02 | 6.13E-03 | -44 | **5.47E-03** | -50 | 4.78E-03 | 6.16E-03 |
| In-113m Indium labelled aerosols substance with fast clearance from lungs | 1.60E-02 | 7.73E-03 | -52 | **7.17E-03** | -55 | 6.41E-03 | 7.93E-03 |
| In-113m Indium labelled aerosols substance with slow clearance from lungs | 2.50E-02 | 2.50E-02 | 0 | **2.58E-02** | 3 | 2.28E-02 | 2.87E-02 |
| In-113m Indium labelled non-absorbable markers, orally administered of fluids | 2.00E-02 | 7.00E-03 | -65 | **7.27E-03** | -64 | 6.73E-03 | 7.80E-03 |
| In-113m Indium labelled non-absorbable markers, orally administered of solids | 2.90E-02 | 7.85E-03 | -73 | **8.35E-03** | -71 | 7.66E-03 | 9.04E-03 |
| K-38 Potassium ultrashort lived | 1.90E-02 | 1.40E-02 | -26 | **1.48E-02** | -22 | 1.35E-02 | 1.60E-02 |
| K-42 Potassium, intravenous | 2.80E-01 | 1.41E-01 | -50 | **1.36E-01** | -51 | 1.24E-01 | 1.48E-01 |
| K-42 Potassium, orally | 3.40E-01 | 2.46E-01 | -28 | **2.34E-01** | -31 | 2.14E-01 | 2.53E-01 |
| K-43 Potassium, intravenous | 2.00E-01 | 1.44E-01 | -28 | **1.37E-01** | -32 | 1.26E-01 | 1.49E-01 |
| K-43 Potassium, orally | 2.20E-01 | 1.50E-01 | -32 | **1.45E-01** | -34 | 1.33E-01 | 1.56E-01 |
| Kr-81m Krypton | 2.70E-05 | 2.67E-05 | -1 | **2.75E-05** | 2 | 2.45E-05 | 3.05E-05 |
| La-140 Lanthanum DTPA, abnormal renal function | 2.60E-01 | 2.42E-01 | -7 | **2.35E-01** | -10 | 2.12E-01 | 2.59E-01 |
| La-140Lanthanum DTPA, normal renal function | 1.50E-01 | 1.02E-01 | -32 | **8.75E-02** | -42 | 7.57E-02 | 9.93E-02 |
| Mg-28 Magnesium | 7.20E-01 | 1.24E-01 | -83 | **1.25E-01** | -83 | 1.12E-01 | 1.39E-01 |
| N-13 Nitrogen Ammonia | 2.00E-03 | 2.35E-03 | 18 | **2.32E-03** | 16 | 2.11E-03 | 2.53E-03 |
| N-13 Nitrogen gas continuous inhalation for 1 hr | 4.30E-04 | 4.20E-04 | -2 | **4.33E-04** | 1 | 3.83E-04 | 4.82E-04 |
| N-13 Nitrogen gas single inhalation with 20 s breathhold | 3.80E-04 | 3.74E-04 | -2 | **3.86E-04** | 2 | 3.41E-04 | 4.30E-04 |
| N-13 Nitrogen gas solution | 4.10E-04 | 4.00E-04 | -2 | **4.12E-04** | 0 | 3.65E-04 | 4.59E-04 |
| N-13 Nitrogen L-glutamate | 3.90E-03 | 3.78E-03 | -3 | **3.38E-03** | -13 | 3.06E-03 | 3.70E-03 |
| Na-22 Sodium, intravenous | 2.60E+00 | 2.93E+00 | 13 | **2.94E+00** | 3 | 2.67E+00 | 3.21E+00 |
| Na-22 Sodium, orally | 2.60E+00 | 2.93E+00 | 13 | **2.94E+00** | 13 | 2.67E+00 | 3.20E+00 |
| Na-24 Sodium, intravenous | 3.20E-01 | 2.90E-01 | -9 | **2.90E-01** | -9 | 2.63E-01 | 3.17E-01 |
| Na-24 Sodium, orally | 3.60E-01 | 3.06E-01 | -15 | **3.08E-01** | -14 | 2.82E-01 | 3.35E-01 |
| O-15 Oxygen Carbon Dioxide, continuous inhalation 1 hr | 3.80E-04 | 3.95E-04 | 4 | **3.98E-04** | 5 | 3.59E-04 | 4.38E-04 |
| O-15 Oxygen Carbon Dioxide, single inhalation with 20 s breathold | 5.10E-04 | 5.34E-04 | 5 | **5.39E-04** | 6 | 4.86E-04 | 5.93E-04 |
| O-15 Oxygen gas, continuous inhalation | 4.00E-04 | 4.33E-04 | 8 | **4.48E-04** | 12 | 4.00E-04 | 4.97E-04 |
| O-15 Oxygen gas, single inhalation | 3.70E-04 | 3.88E-04 | 5 | **4.01E-04** | 8 | 3.58E-04 | 4.45E-04 |
| O-15 Oxygen Monoxide, continuous inhalation for 1 hr | 5.50E-04 | 8.37E-04 | 52 | **8.92E-04** | 62 | 8.16E-04 | 9.68E-04 |
| O-15 Oxygen Monoxide, single inhalation with 20 s breathhold | 8.10E-04 | 1.25E-03 | 54 | **1.33E-03** | 64 | 1.22E-03 | 1.44E-03 |
| O-15 Oxygen water | 1.10E-03 | 9.63E-04 | -12 | **9.33E-04** | -15 | 8.72E-04 | 9.93E-04 |
| P-32 Phosphate | 2.40E+00 | 8.66E-01 | -64 | **8.84E-01** | -63 | 7.94E-01 | 9.73E-01 |
| P-33 Phosphate | 6.60E-01 | 1.25E-01 | -81 | **1.27E-01** | -81 | 1.14E-01 | 1.39E-01 |
| Rb-81 Rubidium | 2.80E-02 | 1.92E-02 | -31 | **1.98E-02** | -29 | 1.80E-02 | 2.16E-02 |
| Rb-81 Rubidium-labelled Denatured erythrocytes | 1.40E-01 | 1.08E-01 | -23 | **5.97E-02** | -57 | 5.40E-02 | 6.55E-02 |
| Rb-82 Rubidium ultrashort lived | 3.40E-03 | 2.39E-03 | -30 | **2.47E-03** | -27 | 2.24E-03 | 2.69E-03 |
| Rb-84 Rubidium | 2.80E+00 | 3.18E+00 | 13 | **3.25E+00** | 16 | 2.98E+00 | 3.51E+00 |
| Rb-86 Rubidium | 3.00E+00 | 1.02E+00 | -66 | **1.06E+00** | -65 | 9.67E-01 | 1.15E+00 |
| S-35 Sulfur Sulphate | 9.00E-02 | 1.00E-01 | 12 | **1.03E-01** | 14 | 9.26E-02 | 1.13E-01 |
| Sc-46 Scandium labelled non-absorbable markers fluids | 1.60E+00 | 9.11E-01 | -43 | **8.37E-01** | -48 | 6.38E-01 | 1.04E+00 |
| Sc-46 Scandium labelled non-absorbable markers solids | 1.70E+00 | 9.42E-01 | -45 | **8.69E-01** | -49 | 6.61E-01 | 1.08E+00 |
| Sc-47 Scandium labelled non-absorbable markers fluids | 7.40E-01 | 4.45E-02 | -94 | **4.14E-02** | -94 | 3.31E-02 | 4.98E-02 |
| Sc-47 Scandium labelled non-absorbable markers solids | 7.60E-01 | 4.58E-02 | -94 | **4.30E-02** | -94 | 3.42E-02 | 5.17E-02 |
| Se-75 Selenium 1-Selenomethionine | 2.50E+00 | 2.86E+00 | 14 | **2.89E+00** | 16 | 2.67E+00 | 3.11E+00 |
| Se-75 Selenium labelled amino acids | 2.20E+00 | 2.39E+00 | 8 | **2.27E+00** | 3 | 2.14E+00 | 2.39E+00 |
| Se-75 Selenium labelled bibe acid SeHCAT | 6.90E-01 | 3.01E-01 | -56 | **3.48E-01** | -50 | 3.16E-01 | 3.79E-01 |
| Se-75 Selenium Selenite | 2.60E+00 | 3.29E+00 | 26 | **3.19E+00** | 23 | 2.93E+00 | 3.44E+00 |
| Se-75 Selenium Selenomethylcholesterol | 1.50E+00 | 1.64E+00 | 9 | **1.55E+00** | 3 | 1.42E+00 | 1.68E+00 |
| Sr-85 Strontium | 7.90E-01 | 7.04E-01 | -11 | **6.85E-01** | -13 | 6.04E-01 | 7.66E-01 |
| Sr-87m Strontium | 6.40E-03 | 6.01E-03 | -6 | **5.97E-03** | -7 | 5.40E-03 | 6.53E-03 |
| Sr-89 Strontium | 3.10E+00 | 9.58E-01 | -69 | **9.73E-01** | -69 | 8.72E-01 | 1.07E+00 |
| Tc-99m Technetium Apcitide | 4.70E-03 | 1.13E-02 | 140 | **1.19E-02** | 153 | 1.10E-02 | 1.29E-02 |
| Tc-99m Technetium DMSA | 8.80E-03 | 1.01E-02 | 14 | **7.49E-03** | -15 | 6.78E-03 | 8.19E-03 |
| Tc-99m Technetium DTPA, (intrathecal administered) cisternal injection | 4.70E-03 | 4.86E-03 | 3 | **4.69E-03** | 0 | 4.25E-03 | 5.12E-03 |
| Tc-99m Technetium DTPA, (intrathecal administered) lumbar injection | 4.80E-03 | 4.77E-03 | -1 | **4.55E-03** | -5 | 4.11E-03 | 4.99E-03 |
| Tc-99m Technetium DTPA, abnormal renal function | 4.60E-03 | 4.52E-03 | -2 | **4.34E-03** | -6 | 3.91E-03 | 4.77E-03 |
| Tc-99m Technetium DTPA, normal renal function | 4.90E-03 | 3.97E-03 | -19 | **3.34E-03** | -32 | 2.85E-03 | 3.83E-03 |
| Tc-99m Technetium EC, abnormal renal function | 4.60E-03 | 4.02E-03 | -13 | **3.67E-03** | -20 | 3.22E-03 | 4.12E-03 |
| Tc-99m Technetium EC, acute renal function | 9.90E-03 | 1.05E-02 | 6 | **7.25E-03** | -27 | 6.44E-03 | 8.07E-03 |
| Tc-99m Technetium EC, normal renal function | 6.30E-03 | 4.63E-03 | -27 | **3.66E-03** | -42 | 3.04E-03 | 4.29E-03 |
| Tc-99m Technetium ECD | 7.70E-03 | 5.97E-03 | -23 | **5.64E-03** | -27 | 5.01E-03 | 6.27E-03 |
| Tc-99m Technetium furifosmin, exercise | 8.90E-03 | 6.57E-03 | -26 | **6.78E-03** | -24 | 6.16E-03 | 7.40E-03 |
| Tc-99m Technetium furifosmin, resting subject | 1.00E-02 | 6.99E-03 | -30 | **7.19E-03** | -28 | 6.53E-03 | 7.85E-03 |
| Tc-99m Technetium gluconate glucoheptonate | 5.40E-03 | 5.88E-03 | 9 | **4.67E-03** | -14 | 4.09E-03 | 5.25E-03 |
| Tc-99m Technetium labelled aerosols substances with fast clearance from lungs | 6.10E-03 | 5.41E-03 | -11 | **5.05E-03** | -17 | 4.39E-03 | 5.70E-03 |
| Tc-99m Technetium labelled aerosols substances with slow clearance from lungs | 1.40E-02 | 1.60E-02 | 14 | **1.67E-02** | 19 | 1.50E-02 | 1.84E-02 |
| Tc-99m Technetium labelled HSA | 6.10E-03 | 9.72E-03 | 59 | **1.02E-02** | 67 | 9.39E-03 | 1.10E-02 |
| Tc-99m Technetium labelled albumin, (intrathecal administered) cisternal injection | 5.00E-03 | 5.70E-03 | 14 | **5.67E-03** | 13 | 5.19E-03 | 6.14E-03 |
| Tc-99m Technetium labelled albumin, (intrathecal administered) Lumbar injection | 5.20E-03 | 6.14E-03 | 18 | **6.14E-03** | 18 | 5.63E-03 | 6.65E-03 |
| Tc-99m Technetium labelled albumin microspheres | 1.00E-02 | 1.15E-02 | 15 | **1.19E-02** | 19 | 1.07E-02 | 1.31E-02 |
| Tc-99m Technetium labelled citrate complex | 6.10E-03 | 5.96E-03 | -2 | **4.93E-03** | -19 | 4.38E-03 | 5.47E-03 |
| Tc-99m Technetium labelled colloids, large colloids early to intermediate diffuse parenchymal liver disease | 1.10E-02 | 1.37E-02 | 24 | **1.11E-02** | 1 | 1.02E-02 | 1.20E-02 |
| Tc-99m Technetium labelled colloids, large colloids intermediate to advance diffuse parenchymal liver disease | 1.30E-02 | 1.52E-02 | 17 | **1.13E-02** | -13 | 1.02E-02 | 1.23E-02 |
| Tc-99m Technetium labelled colloids large colloids normal liver condition | 9.40E-03 | 1.24E-02 | 31 | **1.12E-02** | 19 | 1.05E-02 | 1.20E-02 |
| Tc-99m Technetium labelled colloids, small colloids early to intermediate diffuse parenchymal liver disease | 1.20E-02 | 1.41E-02 | 17 | **1.16E-02** | -3 | 1.06E-02 | 1.25E-02 |
| Tc-99m Technetium labelled colloids, small colloids intermediate to advance diffuse parenchymal liver disease | 1.30E-02 | 1.54E-02 | 18 | **1.15E-02** | -12 | 1.04E-02 | 1.26E-02 |
| Tc-99m Technetium labelled colloids small colloids normal liver condition | 9.70E-03 | 2.18E-02 | 124 | **1.96E-02** | 102 | 1.75E-02 | 2.17E-02 |
| Tc-99m Technetium labelled denatured erythrocytes | 1.90E-02 | 2.58E-02 | 36 | **1.59E-02** | -16 | 1.45E-02 | 1.73E-02 |
| Tc-99m Technetium labelled erythrocytes | 7.00E-03 | 1.06E-02 | 51 | **1.11E-02** | 59 | 1.02E-02 | 1.20E-02 |
| Tc-99m Technetium labelled fibrinogen | 6.20E-03 | 1.01E-02 | 63 | **1.06E-02** | 71 | 9.74E-03 | 1.14E-02 |
| Tc-99m Technetium labelled Heparin | 5.50E-03 | 5.49E-03 | 0 | **5.04E-03** | -8 | 4.46E-03 | 5.62E-03 |
| Tc-99m Technetium labelled HM-PAO | 9.30E-03 | 1.05E-02 | 13 | **9.78E-03** | 5 | 8.95E-03 | 1.06E-02 |
| Tc-99m Technetium labelled human immunoglobulin (HIG) | 7.00E-03 | 9.83E-03 | 40 | **9.42E-03** | 35 | 8.93E-03 | 9.92E-03 |
| Tc-99m Technetium labelled IDA derivatives, normal hepato-biliary conditions | 1.70E-02 | 9.39E-03 | -45 | **9.73E-03** | -43 | 8.93E-03 | 1.05E-02 |
| Tc-99m Technetium labelled IDA derivatives, occlusion of the common bile duct | 7.50E-03 | 1.04E-02 | 39 | **1.06E-02** | 41 | 9.97E-03 | 1.13E-02 |
| Tc-99m Technetium labelled IDA derivatives, occlusion of the cystic duct | 1.40E-02 | 8.82E-03 | -37 | **8.54E-03** | -39 | 7.79E-03 | 9.30E-03 |
| Tc-99m Technetium labelled IDA, derivatives parenchymal liver disease | 9.30E-03 | 7.03E-03 | -24 | **6.60E-03** | -29 | 5.88E-03 | 7.33E-03 |
| Tc-99m Technetium labelled MAA | 1.10E-02 | 1.34E-02 | 22 | **1.40E-02** | 27 | 1.27E-02 | 1.53E-02 |
| Tc-99m Technetium labelled MAG3, abnormal renal function | 6.10E-03 | 4.65E-03 | -24 | **3.92E-03** | -36 | 3.31E-03 | 4.53E-03 |
| Tc-99m Technetium labelled MAG3, acute unilateral renal blockage abnormal kidney | 1.00E-02 | 1.06E-02 | 6 | **7.38E-03** | -26 | 6.53E-03 | 8.22E-03 |
| Tc-99m Technetium labelled MAG3, acute unilateral renal blockage normal kidney | 1.00E-02 | 5.09E-03 | -49 | **4.47E-03** | -55 | 3.90E-03 | 5.04E-03 |
| Tc-99m Technetium labelled MAG3,normal renal function | 7.00E-03 | 5.12E-03 | -27 | **4.00E-03** | -43 | 3.29E-03 | 4.70E-03 |
| Tc-99m Technetium labelled MIBI, exercise | 7.90E-03 | 6.55E-03 | -17 | **6.29E-03** | -20 | 5.80E-03 | 6.78E-03 |
| Tc-99m Technetium labelled MIBI, resting subject | 9.00E-03 | 6.81E-03 | -24 | **6.61E-03** | -27 | 6.14E-03 | 7.07E-03 |
| Tc-99m Technetium labelled monoclonal antibodies fab fragments | 1.70E-02 | 1.32E-02 | -22 | **1.21E-02** | -29 | 1.12E-02 | 1.30E-02 |
| Tc-99m Technetium labelled monoclonal antibodies fab2 fragments | 1.10E-02 | 1.19E-02 | 9 | **1.14E-02** | 4 | 1.06E-02 | 1.23E-02 |
| Tc-99m Technetium labelled monoclonal antibodies, intact antibody | 1.20E-02 | 1.17E-02 | -3 | **1.08E-02** | -10 | 9.95E-03 | 1.16E-02 |
| Tc-99m Technetium labelled non-absorbable markers, orally administered of fluids | 1.90E-02 | 1.06E-02 | -44 | **1.07E-02** | -44 | 9.93E-03 | 1.14E-02 |
| Tc-99m Technetium labelled non-absorbable markers, orally administered of solids | 2.40E-02 | 1.13E-02 | -53 | **1.15E-02** | -52 | 1.07E-02 | 1.24E-02 |
| Tc-99m Technetium labelled phosphates and phosphonates, high bone uptake and/or severely impaired kidney function | 4.70E-03 | 3.85E-03 | -18 | **3.87E-03** | -18 | 3.43E-03 | 4.32E-03 |
| Tc-99m Technetium labelled phosphates and phosphonates, normal uptake and excretion | 5.70E-03 | 4.55E-03 | -20 | **3.99E-03** | -30 | 3.38E-03 | 4.59E-03 |
| Tc-99m Technetium labelled Plasmin | 7.30E-03 | 1.02E-02 | 40 | **9.03E-03** | 24 | 8.30E-03 | 9.76E-03 |
| Tc-99m Technetium labelled platelets (thrombocytes) | 1.20E-02 | 1.75E-02 | 46 | **1.37E-02** | 14 | 1.26E-02 | 1.48E-02 |
| Tc-99m Technetium labelled small colloids, intratumoural adm time to removal 6 h | 1.20E-03 | 1.78E-03 | 48 | **2.24E-03** | 86 | 1.98E-03 | 2.50E-03 |
| Tc-99m Technetium labelled small colloids, intratumoural adm time to removal 18 h | 2.00E-03 | 3.14E-03 | 57 | **3.96E-03** | 98 | 3.49E-03 | 4.43E-03 |
| Tc-99m Technetium labelled tetrofosmin, exercise | 6.90E-03 | 5.54E-03 | -20 | **5.67E-03** | -18 | 5.15E-03 | 6.20E-03 |
| Tc-99m Technetium labelled tetrofosmin, resting subject | 8.00E-03 | 5.92E-03 | -26 | **6.15E-03** | -23 | 5.57E-03 | 6.72E-03 |
| Tc-99m Technetium labelled white blood cells (leukocytes) | 1.10E-02 | 1.28E-02 | 16 | **1.02E-02** | -7 | 9.24E-03 | 1.12E-02 |
| Tc-99m Technetium pertechnegas | 1.20E-02 | 1.52E-02 | 26 | **1.50E-02** | 25 | 1.39E-02 | 1.61E-02 |
| Tc-99m Technetium pertechnetate, intravenous blocking agent given | 4.20E-03 | 4.34E-03 | 3 | **4.02E-03** | -4 | 3.58E-03 | 4.46E-03 |
| Tc-99m Technetium pertechnetate, intravenous no blocking agent given | 1.30E-02 | 1.60E-02 | 23 | **1.58E-02** | 22 | 1.48E-02 | 1.68E-02 |
| Tc-99m Technetium pertechnetate, orally no blocking agent given | 1.40E-02 | 6.48E-03 | -54 | **6.36E-03** | -55 | 5.83E-03 | 6.89E-03 |
| Tc-99m Technetium technegas | 1.50E-02 | 1.79E-02 | 19 | **1.90E-02** | 27 | 1.71E-02 | 2.08E-02 |
| Tl-201 Thallium ion | 1.40E-01 | 1.27E-01 | -10 | **1.02E-01** | -27 | 9.90E-02 | 1.05E-01 |
| Xe-127 Xenon, rebreathing for 10 min | 1.10E-03 | 7.77E-04 | -29 | **7.66E-04** | -30 | 6.98E-04 | 8.34E-04 |
| Xe-127 Xenon, rebreathing for 5 min | 7.10E-04 | 1.22E-03 | 72 | **1.20E-03** | 69 | 1.09E-03 | 1.30E-03 |
| Xe-127 Xenon, single inhalation with 30 s breathhold or intravenous injection with 30 s breathhold | 1.30E-04 | 1.51E-04 | 16 | **1.52E-04** | 17 | 1.38E-04 | 1.66E-04 |
| Xe-133 Xenon, rebreathing for 10 min | 1.10E-03 | 8.23E-04 | -25 | **8.32E-04** | -24 | 7.49E-04 | 9.16E-04 |
| Xe-133 Xenon, rebreathing for 5 min | 7.30E-04 | 1.28E-03 | 75 | **1.30E-03** | 78 | 1.17E-03 | 1.43E-03 |
| Xe-133 Xenon, single inhalation with 30 s breathhold or intravenous injection with 30 s breathhold | 1.80E-04 | 1.97E-04 | 9 | **1.99E-04** | 11 | 1.78E-04 | 2.20E-04 |
| Yb-169 Ytterbium DTPA, (intrathecal administered) cisternal injection | 1.40E-01 | 1.62E-01 | 16 | **1.58E-01** | 13 | 1.42E-01 | 1.73E-01 |
| Yb-169 Ytterbium DTPA, (intrathecal administered) lumbar injection | 1.20E-01 | 1.39E-01 | 16 | **1.35E-01** | 13 | 1.22E-01 | 1.49E-01 |
| Yb-169 Ytterbium DTPA, abnormal renal function | 7.30E-02 | 7.82E-02 | 7 | **7.49E-02** | 3 | 6.73E-02 | 8.26E-02 |
| Yb-169 Ytterbium DTPA, normal renal function | 3.60E-02 | 2.47E-02 | -32 | **2.18E-02** | -39 | 1.89E-02 | 2.47E-02 |
| Zn-62 Zink | 3.50E-01 | 4.57E-02 | -87 | **4.91E-02** | -86 | 4.56E-02 | 5.25E-02 |
| Zn-65 Zink | 8.40E+00 | 1.05E+01 | 25 | **1.11E+01** | 32 | 1.03E+01 | 1.20E+01 |
| Zn-69m Zink | 1.40E-01 | 6.04E-02 | -57 | **6.56E-02** | -53 | 6.12E-02 | 7.00E-02 |
